# Hydrogen‐Bond Structure and Low‐Frequency Dynamics of Electrolyte Solutions: Hydration Numbers from ab Initio Water Reorientation Dynamics and Dielectric Relaxation Spectroscopy

**DOI:** 10.1002/cphc.202000498

**Published:** 2020-09-30

**Authors:** Seonmyeong Kim, Xiangwen Wang, Jeongmin Jang, Kihoon Eom, Simon L. Clegg, Gun‐Sik Park, Devis Di Tommaso

**Affiliations:** ^1^ Center for THz-driven Biomedical System Department of Physics and Astronomy Seoul National University Gwanak-gu 08826 South Korea; ^2^ Advanced Institutes of Convergence Technology Seoul National University Suwon-Si Gyeonggi-do 16229 South Korea; ^3^ School of Biological and Chemical Sciences Materials Research Institute Thomas Young Centre Queen Mary University of London Mile End Road London E1 4NS United Kingdom; ^4^ School of Environmental Sciences University of East Anglia Norwich NR4 7TJ United Kingdom

**Keywords:** ab initio molecular dynamics, dielectric relaxation spectroscopy, electrolyte solutions, hydration number, water orientational dynamics

## Abstract

We present an atomistic simulation scheme for the determination of the hydration number (*h*) of aqueous electrolyte solutions based on the calculation of the water dipole reorientation dynamics. In this methodology, the time evolution of an aqueous electrolyte solution generated from *ab initio* molecular dynamics simulations is used to compute the reorientation time of different water subpopulations. The value of *h* is determined by considering whether the reorientation time of the water subpopulations is retarded with respect to bulk‐like behavior. The application of this computational protocol to magnesium chloride (MgCl_2_) solutions at different concentrations (0.6–2.8 mol kg^−1^) gives *h* values in excellent agreement with experimental hydration numbers obtained using GHz‐to‐THz dielectric relaxation spectroscopy. This methodology is attractive because it is based on a well‐defined criterion for the definition of hydration number and provides a link with the molecular‐level processes responsible for affecting bulk solution behavior. Analysis of the *ab initio* molecular dynamics trajectories using radial distribution functions, hydrogen bonding statistics, vibrational density of states, water‐water hydrogen bonding lifetimes, and water dipole reorientation reveals that MgCl_2_ has a considerable influence on the hydrogen bond network compared with bulk water. These effects have been assigned to the specific strong Mg‐water interaction rather than the Cl‐water interaction.

## Graphical abstract

**Ab initio molecular dynamics** simulations are used to characterize the structural, vibrational, and dynamical properties of water in aqueous magnesium chloride (MgCl_2_) solutions, and to develop a methodology to compute the hydration number (*h*) of aqueous electrolytes from water reorientation dynamics. The application of this protocol gives *h* values of MgCl_2_ solutions in excellent agreement with experimental hydration numbers obtained using GHz‐to‐THz dielectric relaxation spectroscopy.

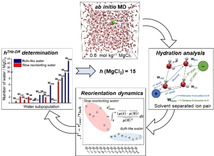

## 1 Introduction

In solution, ions can interact, with a varying degree of strength, with the surrounding water molecules. A key question concerning the effect of salts on any given solution property pertains to the number of water molecules affected. The hydration number (*h*) of an aqueous electrolyte has been loosely understood as the number of water molecules participating in the solvation of the ions and influenced by the presence of the ions.[^1^, ^2^] Knowing the hydration number of aqueous electrolytes is important in several fields. In aqueous electrolyte thermodynamics, various hydration‐based theoretical models have been developed to predict the equilibrium behavior and chemical speciation of natural aqueous solutions (oceans, brines, ground waters and atmospheric aerosols) and industrial systems (ionic liquids, underground contaminants, fluids used for oil and gas processing).[^3^, ^4^, ^5^] These hydration models have mathematical expressions that depend parametrically on the hydration number, here defined as the average number of molecules bound to the compound more strongly than they are bound to other water molecules.^[6]^ Similarly, in the determination of thermodynamic colligative properties (freezing point depression, boiling point elevation, vapor pressure lowering and osmotic pressure), the hydration number refers to the average number of water molecules that are bound sufficiently strongly to the ions so as to be removed from the solvent and become part of the solute.^[7]^ The hydration number is also relevant in the transport of biologically important metal ions at interfaces such as cell membranes,^[8]^ channels,^[9]^ and nanofluidic systems,^[10]^ as tightly bound water increases the apparent ionic size.[^11^, ^12^]

Although the solvation of ions in aqueous solution has been discussed for many years,[^13^, ^14^, ^15^, ^16^, ^17^] there are still several inconsistent estimates for the hydration number of solution electrolytes. For the cesium (Cs^+^) and chloride (Cl^−^) ions, hydration numbers of 9 and 7,^[18]^ respectively, are quoted based on diffraction measurements,^[18]^ which differ, significantly, from the value obtained from colligative properties (freezing point depression data) of CsCl solutions (*h*=0.6±1).^[1]^ MgCl_2_ solutions show the opposite behavior: *h*=14.5 obtained from isothermal compressibility data^[17]^ is much larger than *h*=6 corresponding to the octahedral arrangement of hexa‐aqua magnesium ions (Mg^2+^) deduced from the Mg‐water pair distribution functions generated from X‐ray[^19^, ^20^] and neutron^[21]^ diffraction experiments. Part of the discrepancy lies in the sometimes‐misleading use of the concept of hydration number.

Following Bockris,^[22]^ one should distinguish between coordination numbers and hydration numbers: the coordination number of an ion is the number of water molecules in the immediate vicinity of the ion and depends on the distance between the molecule of water and the ion; the hydration number is based on the dynamical behavior of the water molecules in solution. The values inferred from ion‐water pair distribution function analysis are coordination numbers and have no bearing on the strength of the ion‐water association. For example, both the potassium and magnesium ions have an ion‐water coordination number around six. However, the characterization of the dynamics of the ionic solvation shell using ab initio molecular dynamics (MD) has shown that no water exchanges around Mg^2+^ occur in 50 ps compared to ∼1700 of such events around K^+^.^[23]^ Therefore, two ions with the same coordination number can have very different binding strength with the surrounding water molecules.

Zavitsas proposed a thermodynamic definition of hydration number (*h* ^TMD^) based on the colligative properties of electrolyte solutions as the „dynamic average number of molecules that bind to the solute more strongly than they bind to the other waters”.^[24]^ In this thermodynamic approach, the apparent large deviation from ideal behavior of colligative properties (freezing point, boiling point, vapor pressure, and osmotic pressure) is reduced when the hydration water molecules (*h* ^TMD^) is subtracted from the total number of solvent molecules that are used to compute the mole fraction of the solute.^[25]^ According to this definition, the value of *h* ^TMD^ corresponds to the number of water molecules binding to solute sufficiently strongly as to be removed from the „bulk” solvent. Considering the effect of ions on the dynamics of the water molecules, as originally proposed by Bockris,^[22]^ could provide an approach for the quantification of the hydration number based on well‐defined, molecular‐level behavior of the electrolyte solution.

Terahertz (THz) dielectric spectroscopy measures the dielectric response of material to applied electromagnetic fields. In the GHz‐THz frequency region, the dielectric response of water originates from the reorientation dynamics of the water dipole. From the contribution to the dielectric relaxation mode, the THz‐DR technique is capable of detecting the difference in dynamic behavior between tightly bound and bulk waters.[^26^, ^27^] The hydration number measured from THz‐DR spectroscopy is the average number of moles of water molecules per mole of dissolved salt that no longer participate in bulk‐like reorientation dynamics.^[28]^ This molecular definition of hydration number pertains to „irrotationally bound” waters (the ones tightly bound to the solute) and is consistent with the macroscopic hydration concept proposed by Zavitsas based on thermodynamic considerations.

On the other hand, molecular dynamics (MD) has been extensively used to gain insights into the elusive molecular‐level processes controlling the properties of aqueous electrolyte solutions.[^18^, ^29^] In this simulation technique, the force field used to describe the ion‐water interaction is crucial to obtain an accurate characterization of hydrogen bond (HB) kinetics and reorientation dynamics of water in electrolyte solutions.[^30^, ^31^] A significant contribution to this field was the development of ab initio MD, where forces are computed from the electronic structure,[^32^, ^33^] usually in the framework of density functional theory (DFT),^[34]^ providing the capability of studying non‐additivity effects in the dynamics of ions solvation shells.

**Figure 1:**
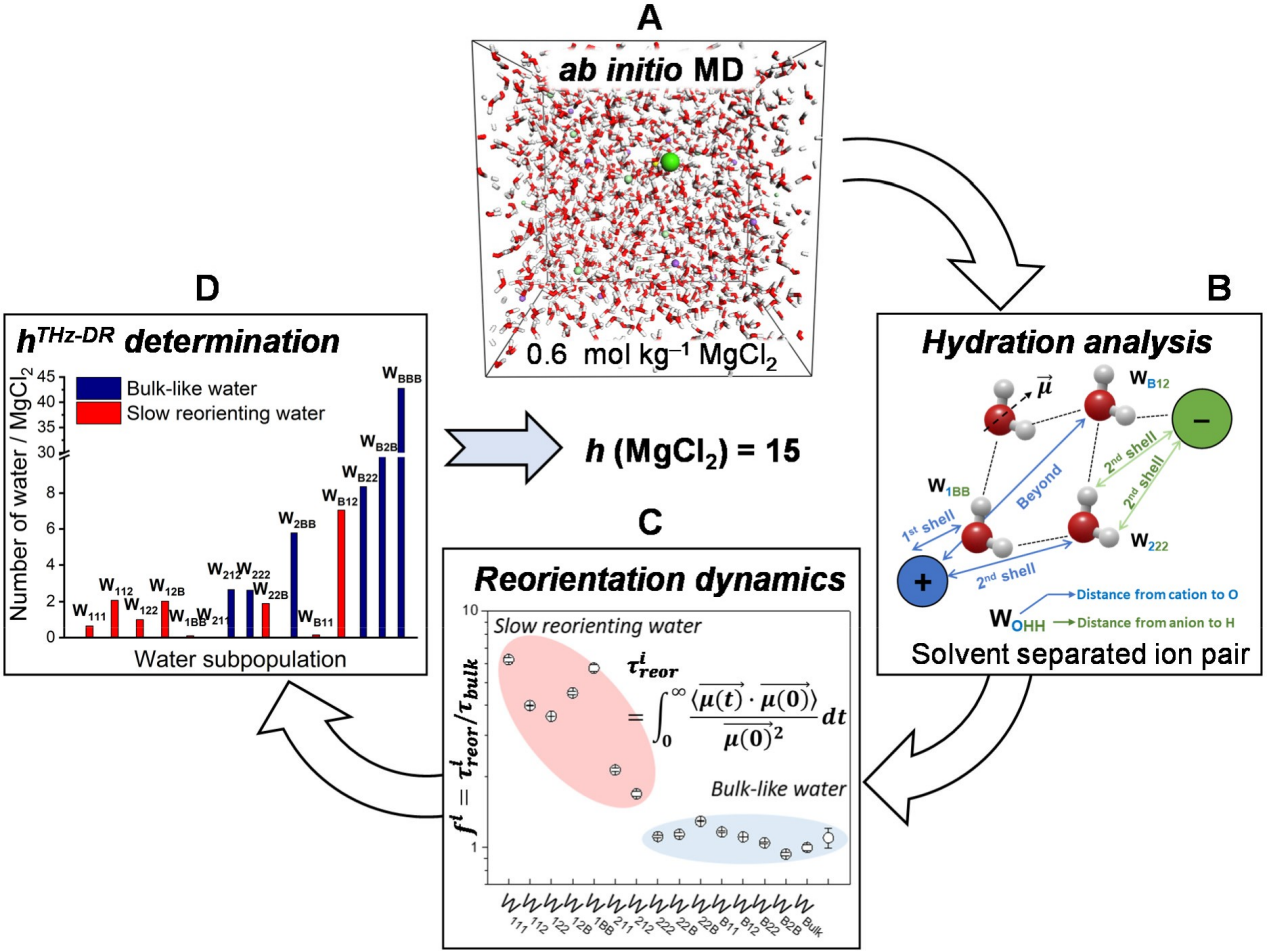
Water reorientation dynamics from *ab initio* MD trajectories and determination of hydration numbers of aqueous electrolytes. The procedure involves generating the time evolution of aqueous electrolyte solution using *ab initio* MD (**A**), from which the water hydration status is determined using the position of H_2_O in the radially varying spherical coordination shell of cations and anions (**B**). The characteristic reorientation time (*τ*_reor_) of each water subpopulation is computed from the bi‐exponential fit of the first‐order Legendre polynomial time correlation function of water dipole vectors (**C**). The hydration number is determined as the number of water molecules, per dissolved salt units, that no longer participate in bulk‐like reorientation dynamics (**D**).

In this work, we present a novel approach to determine the hydration number of an aqueous electrolyte solution by means of *ab initio* calculations of water reorientation dynamics around ions (Figure 1). We report *ab initio* MD simulations of aqueous magnesium chloride solutions, MgCl_2_(aq), with concentrations ranging from 0.1 to 2.8 mol kg^−1^ (solubility of MgCl_2_ in water is 54.3 g 100 mL^−1^ at 20 °C).^[35]^ These simulations have been used to characterize the structure and low‐frequency dynamics of water and to estimate the hydration number of this electrolyte as a function of concentration. We chose Mg^2+^ and Cl^−^ because these ions appear in the composition of seawater and they are also found in biological fluids. The values of *h* for MgCl_2_ obtained from the proposed computational methodology are in good agreement with experimental hydration numbers from THz‐DR measurements of MgCl_2_ solutions, which have also been conducted in this study.

## Methods

### Molecular Dynamics Simulations

#### Ab Initio Molecular Dynamics

Simulations were conducted with the electronic structure code CP2K/Quickstep code, version 4.1.^[36]^ CP2K implements DFT based on a hybrid Gaussian plane wave. The Perdew‐Burke‐Ernzerhof (PBE)^[37]^ generalized‐gradient approximations for the exchange and correlation terms were used together with the general dispersion correction termed DFT‐D3 developed by Grimme at al.^[38]^ to provide a more accurate description of the structure of liquid water.[^39^, ^40^] The Goedecker‐Teter‐Hutter pseudopotentials were used to describe the core‐valence interactions.^[41]^ All atomic species were represented using a double‐zeta valence polarized basis set. The plane wave kinetic energy cut off (E_cut_) was set to 1000 Ry. Table S1 of Supporting Information reports a comparison of the solvation structure of the hydrated cations (isolated ion, no counterions) considered in this study (Mg^2+^, Ca^2+^, Na^+^, K^+^ and Cs^+^) with the data obtained from other simulations and experimental measurements, from which we can deduce that the PBE‐D3 functional with the hybrid Gaussian (DZVP) plane wave (E_cut_=1000 Ry) basis set gives an accurate representation of the first hydration shell structure. The *k*‐sampling was restricted to the *Γ* point of the Brillouin zone. Simulations were carried out with a wave function optimization tolerance of 10^−6^ au. Periodic boundary conditions were applied throughout. The *ab initio* MD simulations were carried out in the canonical constant volume, constant temperature (NVT) ensemble using a Nosé‐Hoover chain thermostat to maintain the average temperature at 300 K with 0.1 ps as the thermostat relaxation time.

#### Simulation Protocol

Details of the electrolyte solutions considered in this study (number of ions and water molecules, average cell lengths and system concentrations) are reported in Table S2 (Supporting Information). First, classical MD simulations of 729 water molecules in the isothermal‐isobaric constant pressure, constant temperature (NPT) ensemble were conducted to generate an equilibrated aqueous solution. The last configuration was then used to generate MgCl_2_(aq) with concentrations ranging from 0.1 to 2.8 mol kg^−1^ by randomly replacing *N* water molecules with *N*/3 Mg^2+^ and 2*N*/3 Cl^−^ ions. As the formation of Mg^2+^/Cl^−^ contact ion pairs (CIPs) has been subject to some debate,[^42^, ^43^] for the 0.63 and 1.30 mol kg^−1^ solutions we generated two set of initial configurations, *with* and *without* CIPs. Each system was subject to 6 ns of classical MD (NPT) simulations to equilibrate the cell volume using the Mg−O and Mg−Cl Lennard Jones potential parameterized by Aqvist^[44]^ together with the SPC/E water model.^[45]^ This combination of force fields has been shown to provide a reasonable description of the structure and dynamic properties of hydrated Mg^2+^.^[46]^ The last configuration was used to initiate *ab initio* MD. Table S3 (Supporting Information) reports the percentages of contact ion pairs (CIP), solvent‐shared ion pairs (SSHIP), and solvent‐separated ion pairs (SSIP) in the 0.1–2.8 mol kg^−1^ MgCl_2_ solutions. The more diluted solutions (0.1–0.6 mol kg^−1^) display a non‐monotonous variation of the contact, solvent‐shared and solvent‐separated ion pairs, which could be related to initial configuration effects because of the slow water dynamics around Mg^2+^ (order of microseconds)^[47]^ and the low number of ions in the simulation box. We have also conducted simulations of pure liquid water, of 0.63 mol kg^−1^ CsCl(aq), and of the hydrated ions Mg^2+^, Ca^2+^, Cs^+^, Na^+^, K^+^ and Cl^−^ (Table S1 in ESI). For the *ab initio* MD simulations of MgCl_2_(aq), each time step required, on average, 45 seconds on 288 cores of the ARCHER UK National Supercomputing Service. The *ab initio* MD simulations reported herein required approximately 890k CPU hours (wall‐clock time × number of processors). Statistics were collected for a period of 20 ps.

## Experimental Details

### Materials

Aqueous MgCl_2_ solutions were prepared by dissolving MgCl_2_ powder in deionized water. MgCl_2_ was purchased from Sigma‐Aldrich with a purity of over 98 % and used without further purification process. High purity deionized water with electrical resistance of 18.2 MΩ cm was prepared by Milli‐Q systems. Solutions were prepared by measuring the weight of MgCl_2_ powder using a balance with a precision of 0.1 mg and dissolving the powder in deionized water by measuring concentration volumetrically (mol kg^−1^). The dielectric constant *ϵ* of liquid sample was determined from measured reflection coefficient *ρs* by applying the bilinear model (Eq. 1), which is recommended for open‐ended coaxial probe experiments.[^48^, ^49^]

(1) $$\epsilon_{s}=\frac{A\rho_{s}+C}{1+B\rho_{s}}$$

The three coefficients A, B and C were determined using three known standards ϵ (deionized water, dimethyl sulfoxide 99.9 % and 2‐propanol) and recording ρ.[^50^, ^51^] Dimethyl sulfoxide 99.9 % and 2‐propanol 99.5 % were purchased from Sigma.

### Dielectric Spectrum

The complex dielectric spectra of aqueous MgCl_2_ solutions were obtained by measuring 1024 equally‐spaced points in the logarithm scale over the frequency range 0.01–110 GHz, using an open‐ended coaxial probe (850070E, Keysight Inc.) connected to Anritsu model MS4647B vector network analyzer (VNA) with 3739C broadband test set. Open‐ended coaxial probe was immersed into a sample solution with a temperature of 25±0.1 °C. Temperature was controlled by the Finepcr ALB 6400 thermostat. Conductivity measurements were conducted using the Toledo Compact Conductivity meter S230 with Cond probe InLab 731‐ISM. Density of all solution was measured by using DMA 500 density meter under accuracy with 0.001 g cm^3^. All experiments were carried out after 2 ml of sample was stabilized for more than one hour in a 25 °C heat bath.

## 2 Results and Discussion

### 2.1 Solvation Structure

**Figure 2:**
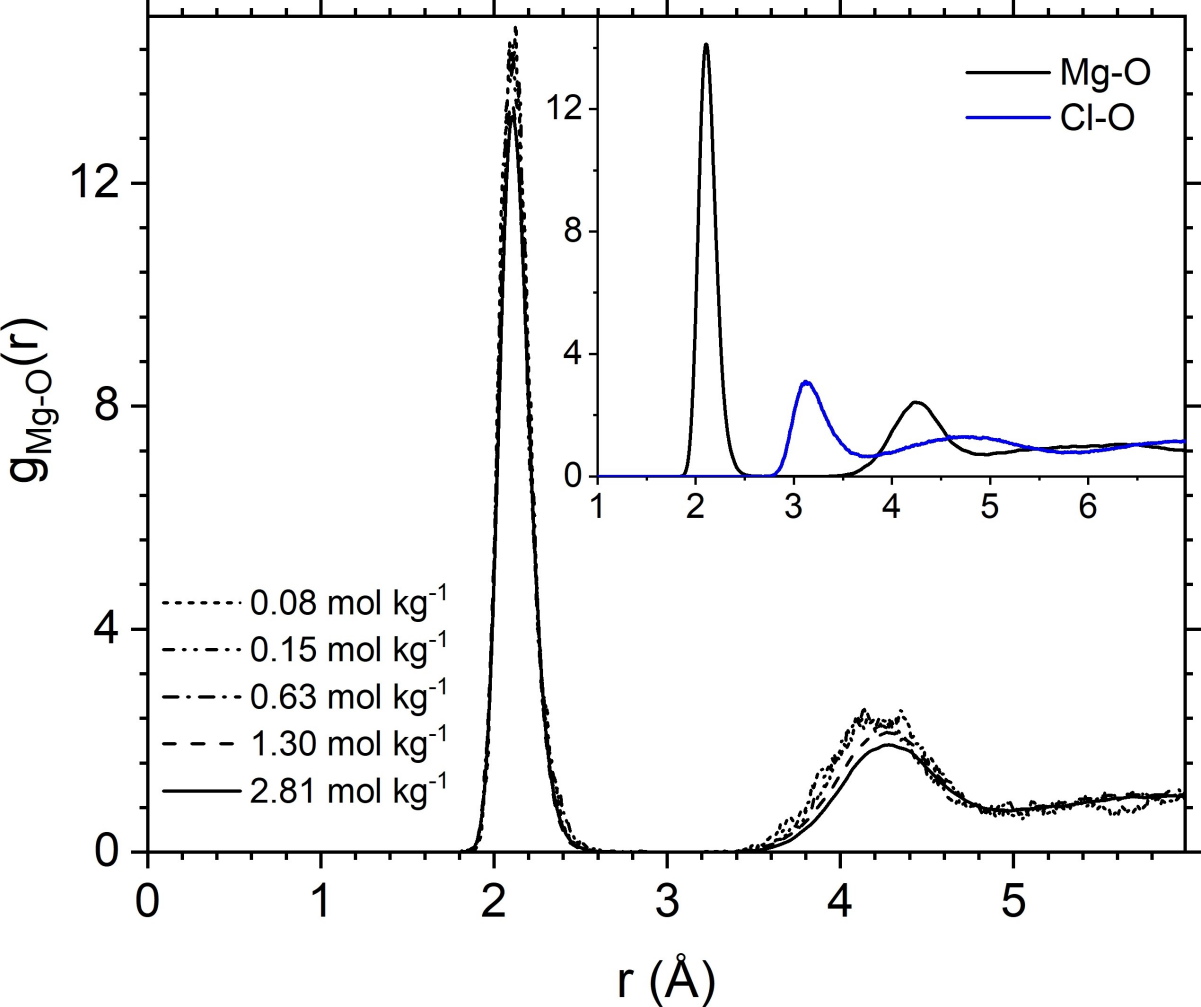
Mg−O radial distribution functions (RDFs) obtained from *ab initio* MD simulations of aqueous MgCl_2_ solutions. *Inset*: Comparison of the Mg−O and Cl−O RDFs obtained from the 0.63 mol kg^−1^ aqueous MgCl_2_ solution.

Experimentally, the first hydration shell Mg−O distance is 2.09±0.04 Å, the average over available diffraction data,^[13]^ and the second shell is in the range of 4.1–4.2 Å.^[14]^ Interatomic Mg‐water distances can be determined from the *ab initio* MD simulations through the generation of the Mg−O radial distribution functions (RDF), *g*(*r*), which represent the probability, relative to a random distribution, of finding Mg and O separated by distance *r* (Figure 2). Key structural properties of the hydration shell of Mg^2+^ obtained from the RDFs (positions, amplitudes, and average coordination number of the first and second hydration shells) are listed Table S4 (ESI). In all solutions, the magnesium ion is characterized by a well‐defined peak at 2.1 Å, which is in excellent agreement with the experimental ranges of equilibrium Mg−O distances (2.09±0.04 Å),^[13]^ and a second widely distributed second shell in the range of 4.1–4.3 Å. The differences between the Mg−O and Cl−O RDF profiles (inset of Figure 2) reflect the rigidity of the hydration shell of Mg^2+^ and the fast exchange dynamics of the water molecules coordinated to Cl^−^: using the „direct” method by Hofer et al. to characterize the dynamics of ionic solvation shell,^[52]^ between 160 and 330 water exchanges every ten ps, depending on the solution concentration and speciation, were accounted around each chlorine ion, to which corresponds a sub‐ps mean residence time of waters in the coordination shell of Cl^−^. In comparison, the intensity of the Mg−O RDF of the MgCl_2_ solutions is zero between the first and second peak because no water exchanges occur around Mg^2+^. The slow kinetics of Mg‐dehydration originates from the high free energy barrier to remove a single water molecule from the first hydration shell of Mg^2+^, as revealed by previous classical MD simulations of hydrated Mg^2+^ (isolated ion, no counterion)[^53^, ^54^, ^55^, ^56^] and MgCl_2_.^[57]^ A recent transition path sampling MD study of the kinetic pathways in the first hydration shell of magnesium concluded that the time spent by water molecules in the first hydration shell of Mg^2+^ is 40 ms.^[58]^

### 2.2 Influence of MgCl_2_ on the Structural Properties of Water

**Figure 3:**
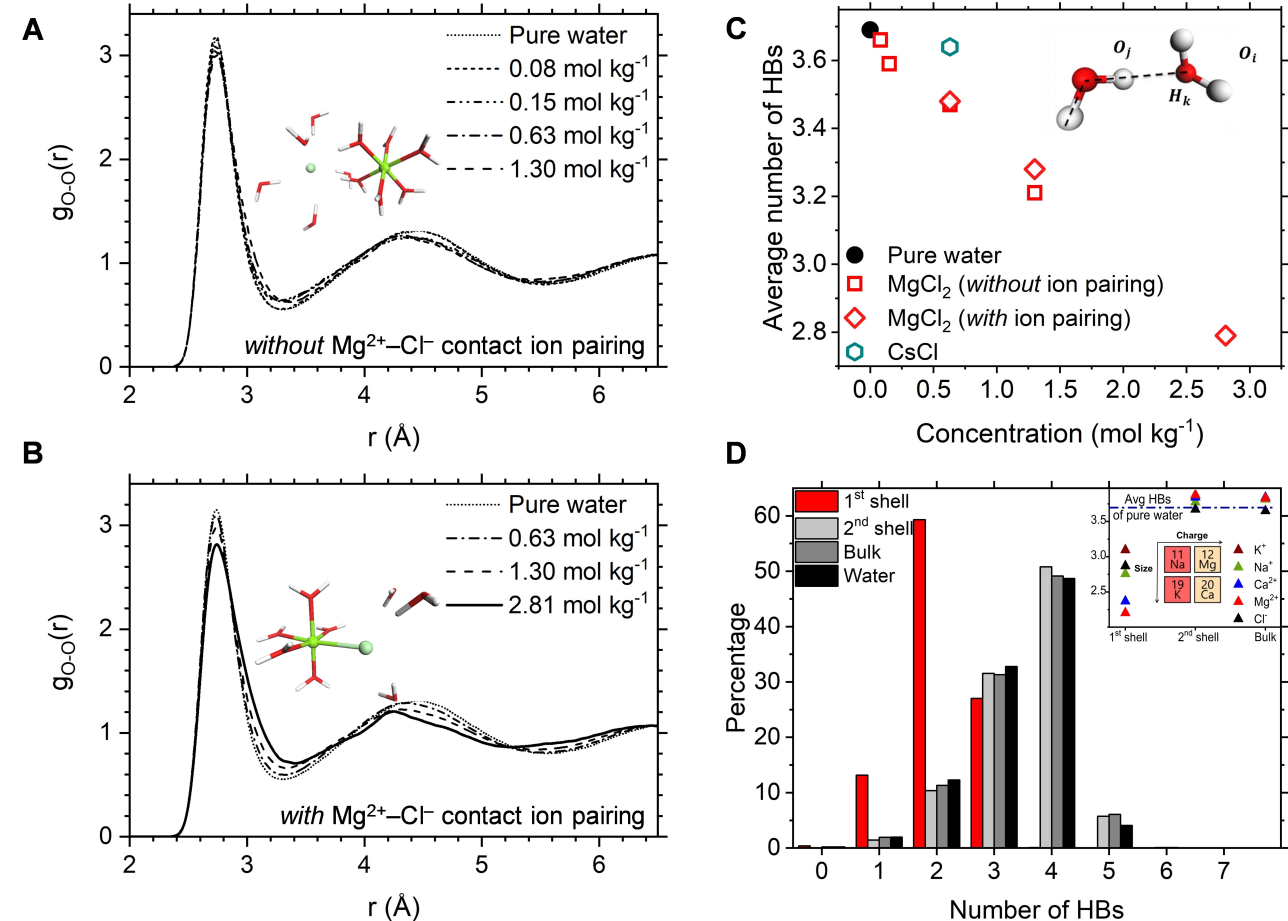
(**A**) Oxygen−oxygen (O−O) radial distribution function, g_O−O_(r), for aqueous MgCl_2_ solutions without Mg^2+^−Cl^−^ contact ion pairing. (**B**) Radial distribution function for aqueous MgCl_2_ solutions with Mg^2+^−Cl^−^ contact ion pairing. (**C**) Average number of hydrogen bonds (HBs) in pure liquid water, aqueous MgCl_2_ solutions and aqueous CsCl solutions. Geometric criteria defining an HB between two H_2_O molecules: ${|O}_{i}-O_{j}|<3.5\;Å$ , ${|O}_{j}-H_{k}|<1.2\;Å$ , ${|O}_{i}-H_{k}|<2.5\;Å$ , $\theta \left(O_{j}O_{i}H\right)<30^{{}^{\circ }}$ ($O_{i}$ is HB acceptor, $O_{j}$ is HB donor). (**D**) Percentage of H_2_O molecules engaging in *n* HBs in the first and second shell of Mg^2+^ and in the bulk. Inset: Average number of HBs in pure water (blue dashed line) and in the solvation shells of hydrated Mg^2+^, Ca^2+^, K^+^, Na^+^ and Cl^−^ ions (isolated ions, no counterions).

The oxygen‐oxygen (O−O) radial distributions (RDF) for pure water and aqueous MgCl_2_ solution, *with* and *without* contact ion pairs, are reported in Figure 3A and Figure 3B. The first and second peak are positioned at 2.74 Å and 4.5 Å, which correspond to the average O−O distance of two hydrogen bonded water molecules and of two water molecules hydrogen bonding to the same water molecule, respectively. The O−O RDF profiles show a progressive rise in the first minimum and lowering of the first and second maxima with increasing concentration, this being associated with a decreasing order in the system. The influence of MgCl_2_ on the water structure is noticeable even at low concentrations (0.15 mol kg^−1^). On the contrary, Gaiduk et al. reported *ab initio* MD simulations of NaCl(aq) where the O−O RDF was very close that that of pure water, even at much higher concentrations (0.9 mol dm^−3^).^[59]^ Similarly, classical MD simulations of KCl(aq) and CsCl(aq) with concentrations ranging from 0.11 to 1.90 mol kg^−1^ did not display significant effects on the water structure.^[60]^

The average number of HBs (*n*_HB_) computed from the *ab initio* MD trajectories of pure water and MgCl_2_ solutions are reported in Figure 3C. We have used a set of geometric criteria where an HB between two water molecules exists if the following distance and angular criteria are satisfied: O−O distance is less than 3.5 Å, H−O distance is less than 2.5 Å, and the O−O−H angle is no more than 30°. Earlier studies considered these geometrical criteria to characterize the statistics and dynamics of hydrogen bonds in pure water,[^61^, ^62^, ^63^] in aqueous electrolyte solutions,[^29^, ^64^, ^65^] in the first hydration shell around hydrated ions,[^66^, ^67^] and at the water‐mineral interface.^[68]^ The values of *n*_HB_ decreases linearly from 3.7 in pure liquid water to 2.8 in the 2.81 mol kg^−1^ MgCl_2_ solution because the fraction of water molecules engaging in two or three HBs increases with the solute concentration (Table S5). A comparison of the HB statistics in the MgCl_2_ solutions obtained from *ab initio* and classical MD simulations is reported in Table S6 and Figure S1 (ESI).

Other chloride‐containing solutions with alkali metal ions such as Cs^+^, which are listed in Table S5, or Na^+^ and K^+[60,64]^ display a similar but less noticeable influence. For example, the average numbers (Figure 3C) and distributions (Table S5) of HBs in the 0.63 mol kg^−1^ MgCl_2_ and CsCl solutions shows that the influence of MgCl_2_ on the HB network is significantly more pronounced than CsCl. Therefore, the presence in solution of Mg^2+^ causes the observed, large perturbations to the water HB network; the distribution of HBs in the Mg^2+^ first hydration shell (Figure 3D**)** has large deviations from bulk behavior, with the waters directly coordinated to Mg^2+^ being mostly hydrogen‐bonded to only two other molecules. The inset of Figure 3D compares the average number of HBs for the water molecules in the first and second hydration shell of the divalent cations Mg^2+^ and Ca^2+^, of the monovalent cations K^+^, Na^+^, and Cs^+^, and of the anion Cl^−^. In the first coordination shell of the ions, the *n*_HB_ values is the lower than in the bulk but this effect is particularly strong for the water molecules directly coordinated to Mg^2+^ (**n**_HB_=2.25). This further confirms that the influence of MgCl_2_ on the water‐water HB network is due to the specific Mg‐water interaction. For all ions, the average number of HBs for the water molecules in the second hydration shell converges to the of „bulk” water (beyond the first and second shells). However, small differences can be observed between the values of „bulk” water and pure liquid water, which are likely due to finite size effects (size of the simulation box). In fact, *ab initio* MD simulations of one Ca^2+^ ion in 124 H_2_O (50 ps) gives *n*_HB_=3.72 for „bulk” water, which is very close to that obtained for pure liquid water (3.7). The results in the inset of Figure 3D also confirms the long‐range effects of ions on the water‐water HB network discussed by Gaiduk and Galli.^[69]^

### 2.3 Influence of MgCl2 on the Low‐Frequency Water Dynamics

#### 2.3.1 Hydration‐Shell Vibrational Density of States

**Figure 4:**
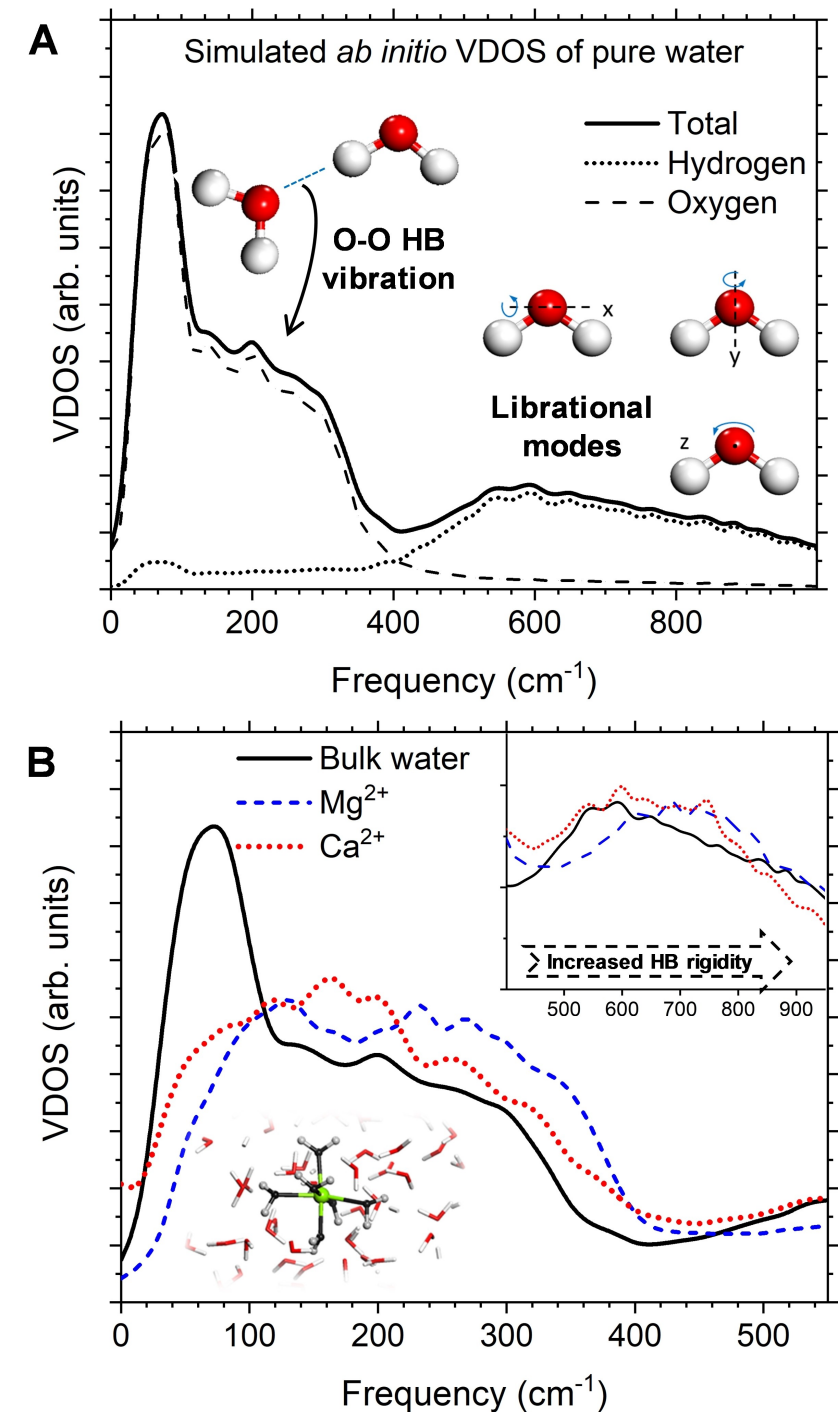
Vibrational density of states (VDOS) from *ab initio* MD. (A) VDOS of pure water showing the vibrational (HB stretch) and librational bands. (B) Comparison of the VDOS of pure water and of the water molecules in the first hydration shell of Mg^2+^ and Ca^2+^. The first shells were defined by the position of the first minimum of the Mg−O (3.0 Å) and Ca−O (3.2 Å) RDFs.

Further insights into the effect of ions on the HB network were obtained from the vibrational density of states (VDOS) of the excitation spectrum of water (Figure 4), which was computed from the ab initio MD trajectories as the sum of the Fourier transform of the velocity‐autocorrelation function (VACF) of the oxygen and hydrogen atoms:^[70]^

(2) $$f\left(\omega \right)=ℱ\left(\gamma \left(t\right)\right)=ℱ\left(\frac{\left\langle \sum {\vec{v}}_{i}\left(0\right){\vec{v}}_{i}\left(t\right)\right\rangle }{\left\langle \sum {\vec{v}}_{i}\left(0\right){\vec{v}}_{i}\left(0\right)\right\rangle }\right)$$

where ***ν**_i_* is the velocity vector of O or H atoms in the *i*‐th water molecule, and the sum is taken over all atoms in the system. In the low frequency region (0–1200 cm^−1^), the spectrum (Figure 4A) has a peak at 50 cm^−1^ corresponding to the O−O−O bonding intermolecular motion, at ∼250 cm^−1^ corresponding to the O−O intermolecular stretching, and a band between 300 and 1200 cm^−1^ that has been assigned to librational modes, hindered rotational motions of water about the three principal inertia axis of free water.^[71]^ These vibrational (HB stretch) and librational bands are very sensitive to the specific ion‐water interactions and ordering of water.[^72^, ^73^] We investigated the local effect of the metal cations to the excitation spectrum of water by restricting the averages in Eq. 2 to the water molecules belonging to the first hydration shell of Mg^2+^ and Ca^2+^, which were defined by the position of the first minimum in the ion‐water radial distribution functions. A previous *ab initio* MD study of the spectroscopic properties of water around small hydrophobic solutes did not show significant differences with respect the bulk signal,^[74]^ but Figure 4B displays the appearance of clear fingerprints of the ionic solvation shell in the libration region of the VDOS. Moreover, the HB peaks and the librational part of *in‐shell* water of Mg^2+^ is more structured and shifted to higher‐frequency modes compared to Ca^2+^. This suggests a stronger water‐water HB network around the magnesium ion. In the first hydration shell of these two cations, the bending H−O−H mode is also blue‐shifted by approximately 50 cm^−1^ compared to bulk water (ca. 1650 cm^−1^), whereas less clear conclusions can be drawn from the O−H stretching region between 2500 and 4000 cm^−1^ of the VDOS (Figure S2 in ESI). An X‐ray spectroscopy study of MgCl_2_(aq) by Techer and co‐workers assigned the distortions of the pre‐, main, and post‐edge of the X‐ray absorption spectra on the oxygen K‐edge in the vicinity of the ions to the strengthening of the HBs in the solvation shell around Mg^2+^.^[75]^ Our *in shell* VDOS analysis agrees with this assignment. Further support to this conclusion is provided by the analysis of the average water‐water distance between the water molecules around Mg^2+^ (3.0 Å), which significantly shorter than Ca^2+^ (3.3 Å) (Figure S3 in ESI).

#### 2.3.2 Hydrogen‐Bond Kinetics

According to the approached proposed by Rapaport,^[76]^ the dynamics of breaking and making of hydrogen bonds can be quantified in terms of the continuous HB time correlation function (TCF), **S**_HB_(**t**), which gives the probability that a pair, **i** and **j**, remains continuously hydrogen‐bonded from 0 to **t**:[^29^, ^77^]

(3) $$S_{HB}\left(t\right)=\frac{\left\langle h_{ij}\left(0\right)\cdot H_{ij}\left(t\right)\right\rangle }{\left\langle {h_{ij}\left(0\right)}^{2}\right\rangle }$$

**Figure 5:**
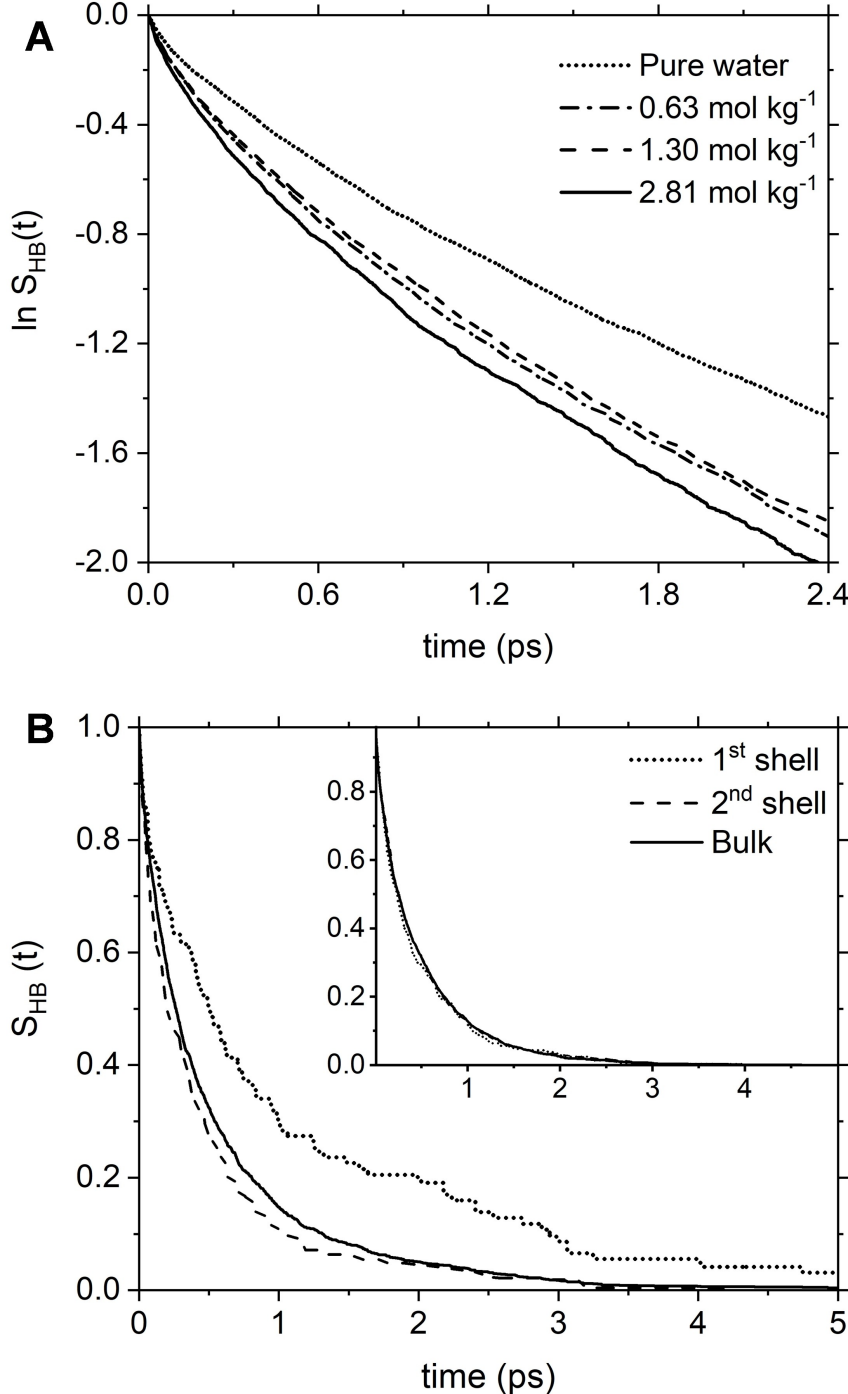
The time dependence of the natural logarithm of the continuous hydrogen bonding (HB) time correlation functions *S*_HB_ (*t*) for aqueous MgCl_2_ solutions at different concentrations. (**A**) Solutions *without* Mg^2+^−Cl^−^ contact ion pairing. (**B**) Solutions *with* Mg^2+^−Cl^−^ contact ion pairing. (**A**) Profiles of *S*_HB_(*t*) for pure water (dotted line) and MgCl_2_ solutions (dashed and solid lines). (**B**) Profiles of *S*_HB_(*t*) for the water molecules in the first and second shell of Mg^2+^ and Cl^−^ (inset) compared with bulk behavior.

The hydrogen bond population variables *h_ij_*(*t*) and *H_ij_*(*t*) in Eq. 3 are defined in the following way: *h_ij_*(*t*)=1 when a tagged water pair is HB at time *t* and *h_ij_*(*t*)=0 otherwise; *H_ij_*(*t*)=1 if the tagged water pair remains *continuously* HB in the time interval [0, *t*] and *H_ij_*(*t*)=0 otherwise. To construct this correlation function, we have used the geometrical criteria of HB.^[64]^ The brackets in Eq. 3 denote average over all water pairs in the solution. We obtained well convergent *S_HB_*(*t*) profiles by using multiple time origins and overlapping intervals [0, *t*] of time length equal to 11 ps. The detailed protocol used to compute TCFs is explain in ESI (Calculation of time correlation functions and Figure S6–S9) and is based on the procedure outlined by Leach.^[78]^ The average HB lifetime, *τ*_HB_, can be determined from the integration of Eq. 3. Values of *τ*_HB_ equal to 1.56 ps and 1.31 ps were obtained from simulation boxes containing 729 and 64 water molecules, respectively, which are within the experimental range of 0.5–1.7 ps.^[79]^ These results support the *ab initio* MD methodology used in this study to characterize the low‐frequency water dynamics. Figure 5A compares *S*_HB_(*t*) profiles obtained for pure liquid water and MgCl_2_(aq) *with* Mg^2+^−Cl^−^ contact ion pairs in solution; the analysis of the solutions *without* ion pairing gave very similar results. The HB dynamics are faster in MgCl_2_ solutions (faster decay of *S*_HB_(*t*) profiles) than in pure water and accelerates with the solute concentration. This is confirmed by the average HB lifetimes in aqueous MgCl_2_ solutions, which are lower than pure water; the fastest dynamics of HB making/breaking is observed for the 2.81 mol kg^−1^ MgCl_2_ solution *with* CIPs (Table S7 in ESI). Therefore, the presence of Mg^2+^ and Cl^−^in solution weakens the water‐water strength of pairing. Further insights can be obtained from the characterization of the HB dynamics of the water molecules in the first hydration shells of Mg^2+^ and Cl^−^. In Figure 5B, the *S*_HB_ (*t*) profiles of the first hydration shell of Mg^2+^ has a slower decay (slower HB dynamics) than bulk water and the *S*_HB_ (*t*) profiles of the second hydration shells of Mg^2+^ has faster decay (faster HB dynamics) than bulk water. The effects of Mg^2+^ on the surrounding water molecules follow, therefore, the ionic hydration model proposed by Frank and Wen:^[80]^ in the innermost region the water molecules are tightly bound to Mg^2+^ and exhibit lower HB dynamics than those in pure water; in the second region the magnesium ion induces a „structure breaking effect“, wherein disruption of the hydrogen bonding network enhances the mobility of the water molecules.^[81]^ Dissolved Mg^2+^ ions have a long‐range effect, which goes beyond the first hydration shell. In comparison, the *S*_HB_(*t*) profiles of the first and second hydration shells of Cl^−^ overlap with that of bulk water (Figure 5B) suggesting that the chloride ion does not have short‐ or long‐range effects on the HB dynamics of water. The computed values of the HB relaxation time of the solvent molecules in the first (1.35 ps) and second (1.25) shell of chloride, and in the bulk of the solution (1.28 ps) confirm this observation (Table S7 in ESI). The different influence of Mg^2+^ and Cl^−^ on the HB dynamics of the surrounding water molecules should be linked to the hydration numbers of these two ions, which have estimated values of *h*=15 for Mg^2+^ and *h*=0 for Cl^−^, according to (thermodynamic) colligative^[1]^ and isothermal compressibility data.^[17]^ The long‐range effects of Mg^2+^ beyond its first shell also explains why the hydration number does not correspond to the coordination number (6).

#### 2.3.3 Water Reorientation Dynamics

**Figure 6:**
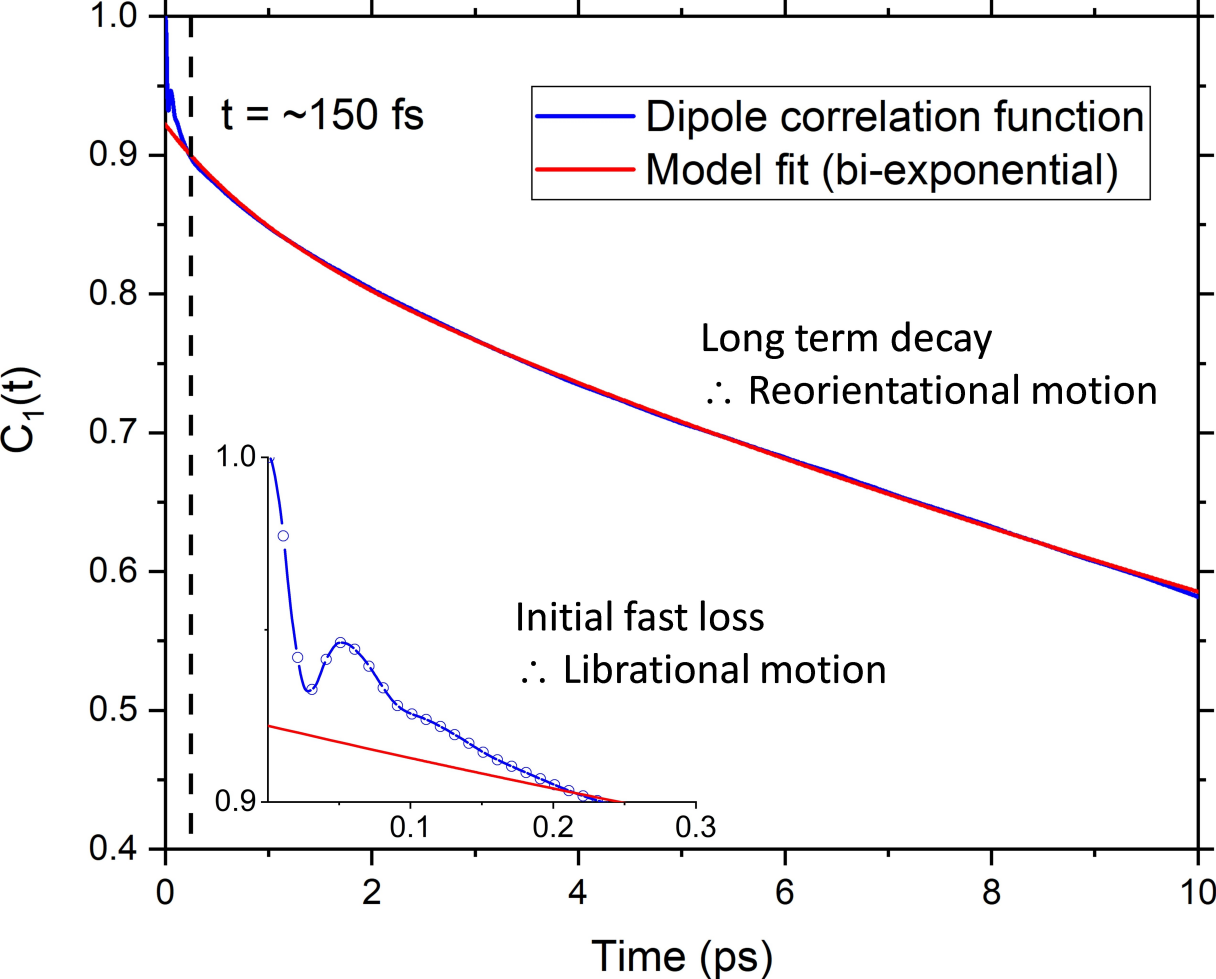
Orientation time correlation function C_1_(t) obtained from ab initio MD simulations of pure liquid water. The early stage of fast loss of correlation is caused by librational motion. The long‐term decay is due to reorientational motion and is fitted by a bi‐exponential function a⋅exp(‐t/τ_1_)+b⋅exp(‐t/τ_2_).

Rotational motion of water molecules plays a crucial role in the breaking and making of HB (more strongly hydrogen‐bonded water molecules reorient more slowly).[^82^, ^83^] We have quantified the rotational relaxation of the water dipole by computing the first‐order Legendre polynomial time correlation function:^[84]^

(4) $$C_{1}\left(t\right)=\;\frac{\left\langle \vec{\mu }\left(0\right)\cdot \vec{\mu }\left(t\right)\right\rangle }{{\vec{\mu }\left(0\right)}^{2}}$$

where ***μ***(0) and ***μ***(*t*) are the unit vectors defining the orientation of the dipole moment of a water molecule at times 0 and *t*, respectively. The average in Eq. 4 was computed over all water molecules in the solution, using multiple time origins and overlapping intervals^[0,*t*]^ of equal time length (*t*=16 ps) (Figures S4–S7 in ESI).^[78]^ In Figure 6, the **C**_1_(**t**) function starts at 1 and decays asymptotically to zero because of the random and isotropic orientation of the water molecules in solution. The early stages of fast loss of correlation is caused by librational motion, whereas the long term decay is due to reorientational motion and can be fit by the bi‐exponential function *a*⋅exp(−*t*/*τ*_1_)+*b* ⋅ exp(−*t*/*τ*_2_).^[84]^ The relaxation time associated with the reorientational motion, *τ*_reor_, is given by the weighted average of the fitting parameters *τ*_1_ and *τ*_2_:

(5) $$\tau_{reor}=\frac{a\cdot \tau_{1}+b\cdot \tau_{2}}{a+b}$$

**Figure 7:**
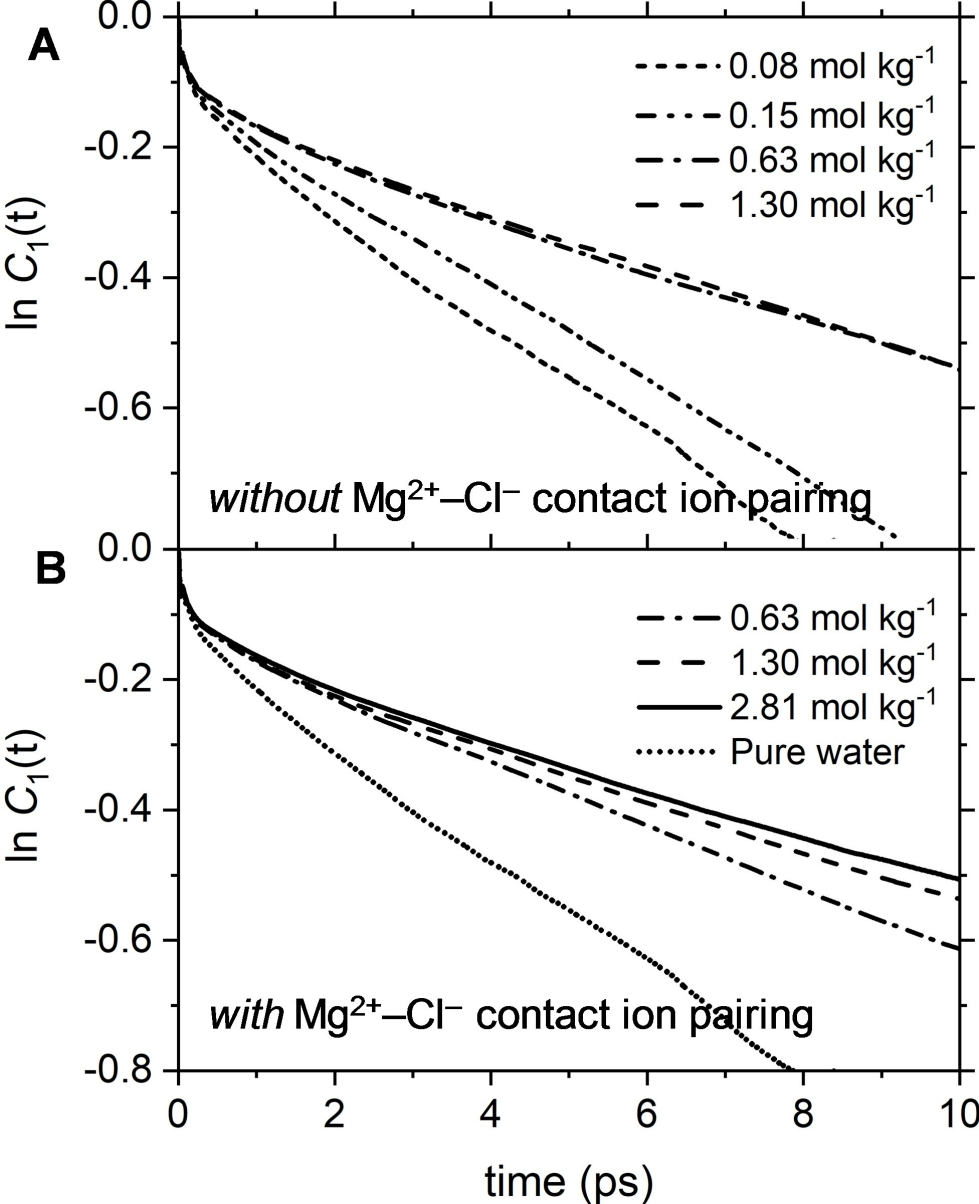
The time dependence of the natural logarithm of the orientation time correlation function *P*1(*t*) for aqueous MgCl_2_ solutions at different concentrations. (**A**) Solutions *without* Mg^2+^−Cl^−^ contact ion pairing. (**B**) Solutions *with* Mg^2+^−Cl^−^ contact ion pairing. (**C**) Values of the first order orientational relaxation times, τ_reor_, as a function of the solution concentration.

The biexponential fitting curve gives a more accurate estimate of the relaxation time associated with the water reorientation process because it removes the contribution from the water librational dynamics. It is also important to note that the single‐water relaxation times computed from the integration of the first‐order Legendre polynomial‐time correlation functions cannot be directly compared with reorientation times obtained experimentally using DRS. To realize such comparison the TCF of the *total* system dipole correlation should be considered by computing the auto‐ and cross‐correlation terms, as done by Sega and Schroder,^[85]^ and Zarzycki and Gilbert.^[86]^ The concentration‐dependent time correlation profiles, *C*_1_(*t*), of the MgCl_2_ solutions are reported in Figure 7. Compared to pure water, the dipole reorientation dynamics is slower in MgCl_2_(aq) and decreases with the solution concentration. The *C*_1_(*t*) profiles are also significantly influenced by the type of ion pairs present in solution; the 0.63 and 1.30 mol kg^−1^ solution *without* CIPs have very similar water reorientation behaviour (Figure 7A), whereas significant differences can be observed in the solutions *with* CIPs (Figure 7B). The correlation function *C*_1_(*t*) is, therefore, sensitive to solution speciation and could provide insights into the cooperative effect of Mg^2+^ and Cl^−^ ions on the water dipole reorientation dynamics. In the next section, changes compared to bulk behavior will be used to determine the hydration numbers of aqueous MgCl_2_ solutions as a function of concentration.

### 2.4 Hydration Numbers from Water Reorientation Dynamics

#### 2.4.1 Cooperative Hydration Model

**Figure 8:**
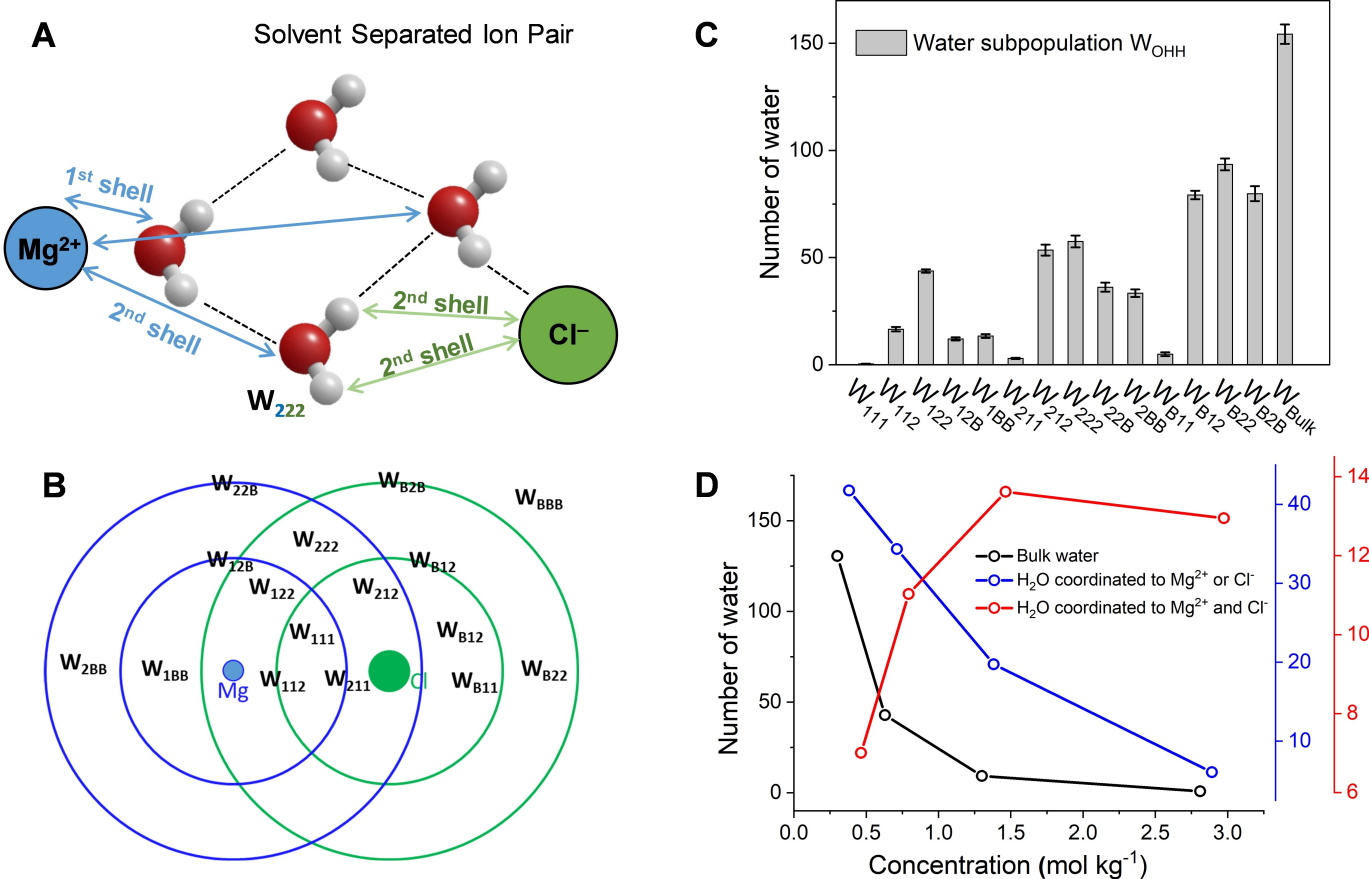
(**A**) Definition of subpopulations near a solvent separated Mg^2+^−Cl^−^ ion pair showing the categorization of a water molecule in the W_222_ subpopulation: the oxygen is in the 2^nd^ coordination shell of Mg^2+^ and both hydrogen atoms are in the 1^st^ coordination shell of Cl^−^. (**B**) Overlapping of the 1^st^ and 2^nd^ coordination spheres of the Mg^2+^ and Cl^−^for a solvent separated ion pair showing the water. (**C**) Distribution of molecules among the subpopulations for the 1.3 mol kg^−1^ MgCl_2_ solution. Subpopulation analysis conducted at each time step (1 fs) using four different time origins. Standard errors computed from the variations of block averages, each lasting 5 ps. (**D**) Number of water molecules in the bulk (black), coordinated to either Mg^2+^ or Cl^−^ (blue), or coordinated to both Mg^2+^ or Cl^−^ (red). Values normalized to MgCl_2_ units.

Speciation analysis of the aqueous MgCl_2_ solutions (Table S2 in ESI) shows that Mg^2+^ and Cl^−^ ions are mainly present as free ions only in the most dilute solutions (0.08 and 0.15 mol kg^−1^). Otherwise, they form contact, solvent shared, and solvent‐separated ion pairs. As cooperative effects of ions in the aqueous electrolyte can induce specific changes to the water dynamics,[^87^, ^88^] we have implemented a cooperative hydration model to categorize the water molecules in MgCl_2_(aq) in different subpopulations (Figure 8A) and characterize the dynamic properties of water depending on the relative position from the ions. The hydrogen and oxygen atoms of each water molecule in the solution are labelled O_*a*_H_*b*_H_*c*_, where *a*, *b*, *c*=1, 2, and B, depending on the position from the Mg^2+^ and Cl^−^ ions; the subscript is set to 1 when the oxygen or hydrogen atom is in the first coordination shell of the nearest ion, to 2 when the oxygen or hydrogen atom is in the second coordination shell of the nearest ion, and B when it is beyond the second shell. Assignments were made by comparing the distance between O_*a*_ and the nearest magnesium ion with the positions of the first and second minima of the Mg−O RDFs (Figure 2), and the distance between H_*b*_ (or H_*c*_) and the nearest chlorine ion with first and second minima of the Cl−H RDF (Figure S9 in ESI). The application of these criteria to categorize water molecules in MgCl_2_ solutions is based on the semi‐rigid hydration scheme, where water dynamics are „locked” in two directions: the orientation of the water dipole is mainly affected by cations; the O−H orientation is mainly affected by anions.^[87]^ Previous experimental and simulation work provide evidence for orientation ordering of water in extended hydration shells around the ion.[^89^, ^90^, ^91^, ^92^] Using this approach, the water molecules in the solution can be classified into 18 subpopulations, W_*abc*_, where *a*, *b*, *c*=1, 2, B. For examples, W_112_ refers to the subpopulation O_1_H_1_H_2_ of molecules having the oxygen in the first coordination shell of Mg^2+^, one hydrogen atoms in the first coordination shell of Cl^−^, and the other hydrogen in the second shell of Cl^−^. The number of water molecules in W_11B_, W_21B_, and W_B1B_ are zero because these subpopulations correspond to molecules where the O−H bond is dissociated, that is one hydrogen is in the first shell and the other hydrogen in the bulk.The computational procedure used to categorize the water molecules in MgCl_2_(aq) into different water subpopulation is presented in ESI, together with a detailed analysis of the process of water „exchange” between different subpopulations. Figure 8C reports the distribution of water molecules among the fifteen subpopulations in the 1.3mol kg^−1^ MgCl_2_ solution. The subpopulation analysis has been conducted at each time step using four (non‐overlapping) simulation blocks each lasting 5 ps. The error bars in Figure 8C are <1 % because of the small variation of the average number of water molecules in each subpopulation during the four consecutive time blocks **(**Figure S13 in ESI). From this population distribution is also possible to evaluate the number of water molecules that are in the bulk (or free water), coordinated to one ion, or coordinated to both Mg^2+^ and Cl^−^ (Figure 8D). The fraction of bulk and single‐coordinated ions per number of Mg^2+^ decreases as the MgCl_2_ concentration increases and beyond 1.3 mol kg^−1^ most molecules are coordinated to both ions. This makes cooperative ionic effects important in most solutions.

#### 2.4.2 Reorientation Dynamics in Different Subpopulations

For each water subpopulations, the orientation time correlation function was computed by tracking the dipole vectors of the water molecules belonging to that specific subpopulation. We used 1024 time origins to generate well convergent *C*_1_(*t*) profiles from which we computed the reorientation relaxation time of each water subpopulation. We define the *retardation factor* as the ratio between the reorientation relaxation time of the subpopulation *i* and bulk water:

(6) $$f_{i}=\frac{\tau_{W_{OHH}}^{i}}{\tau_{W_{bulk}}}$$

**Figure 9:**
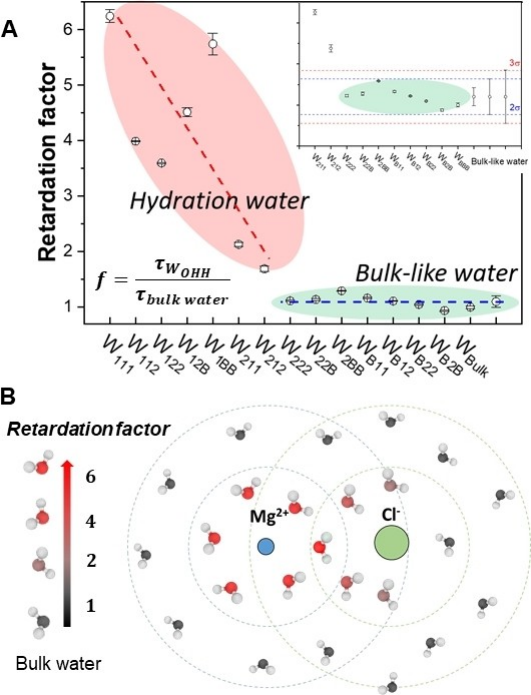
Retardation factor of each water subpopulation. **(A)** Retardation factor for the reorientation relaxation time of the water subpopulations in the 2.81 mol kg^−1^ solution. Standard errors computed from the variations of block averages, each lasting 5 ps, using four different time origins. *Inset*: statistical approach used to classify bulk‐like and hydration water based on the Empirical Rule (68‐95‐99.7 rule): if the population of a statistical data set has a normal distribution with population mean and standard deviation (*σ*), than about 99.7 % of the values lie within 3 standard deviations of the mean. **(B)** Representation of the slow relaxation dynamics (red area) for the subpopulations of molecules in the first‐ or second shell of Mg^2+^ and in first coordination shell of Cl^−^.

The retardation factors for the subpopulations in the 2.81 mol kg^−1^ MgCl_2_ solution are reported in Figure 9A. The *inset* of this figure presents the statistical approach we have adopted to classify bulk‐like and hydration water, which is based on the Empirical Rule, also known as the 68–95–99.7 rule: for a subpopulation to be classified as bulk‐like water the retardation factor must lie within 3*σ* of the mean value of bulk‐like water (green domain in Figure 9A). Water molecules in W_222_, W_22B_, W_B11_, W_B12_, W_B22_, and W_B2B_ (*f*≈1) are within the 2*σ* deviation and are classified as bulk‐like water. On the other hand, a slow relaxation dynamics (*f* values 2–6 times larger compared to the bulk) is observed for water molecules that are in the first coordination shell of Mg^2+^ (subpopulations W_111_, W_112_, W_122_, W_12B_, W_1BB_) or in the second coordination shell of Mg^2+^ and first coordination shell of Cl^−^ (W_211_, W_212_) (Figure 9B). In particular, W_211_ and W_212_ are distributed well beyond the 3*σ* deviation (even larger than 4*σ*) and contribute, therefore, to the hydration number of MgCl_2_(aq). The *C*_1_(*t*) profiles of the water molecules in the first and second coordination shells of hydrated Mg^2+^ and Cl^−^ (isolated ion, no counterions) confirm the long‐range effect of Mg^2+^ and short‐range effect of Cl^−^ on the reorientation water dynamics (Figure S14 in ESI). This result also agrees with a theoretical analysis on individual water entropy around ions, which showed that the rotational entropy reduction of the first solvation shell water molecules near Cl^−^ is almost half compared to that around Mg^2+^.^[93]^ The retardation of the water dipole reorientation near ions, including Mg^2+^,^[94]^ has also been discussed in the „jump model” by Stirnemann et al. to explain the long‐ and short‐range effects of Mg^2+^ on the orientation time correlation function. Our finding that subpopulations such as W_211_ and W_212_ in MgCl_2_(aq) have a slow reorientation dynamics compared to bulk behaviour supports previous investigations of electrolyte solutions reporting on the long‐range effects of ions, beyond their first hydration shell.[^87^, ^95^, ^96^] The combined THz absorption spectroscopy (frequency region 50–640 cm^−1^) and classical MD study of MgSO_4_ solutions by Vila Verde et al.^[96]^ suggests that the reorientational dynamics of the water molecules between two ions in the solvent shared configuration is slowed down, via a cooperative, supra‐additive, effect. Also, the hydration number of Mg^2+^ deduced from THz dielectric relaxation and THz absorption spectroscopy is well above the number of water molecules in the first hydration shell of Mg^2+^ (6),^[87]^ which again suggests that some portion of water molecules beyond the first hydration shell of Mg^2+^ have different physical property (such as vibrational absorption or reorientation dynamics) compared to bulk water.

#### 2.4.3 Hydration Numbers of Aqueous MgCl_2_ from water Reorientation Dynamics

**Figure 10:**
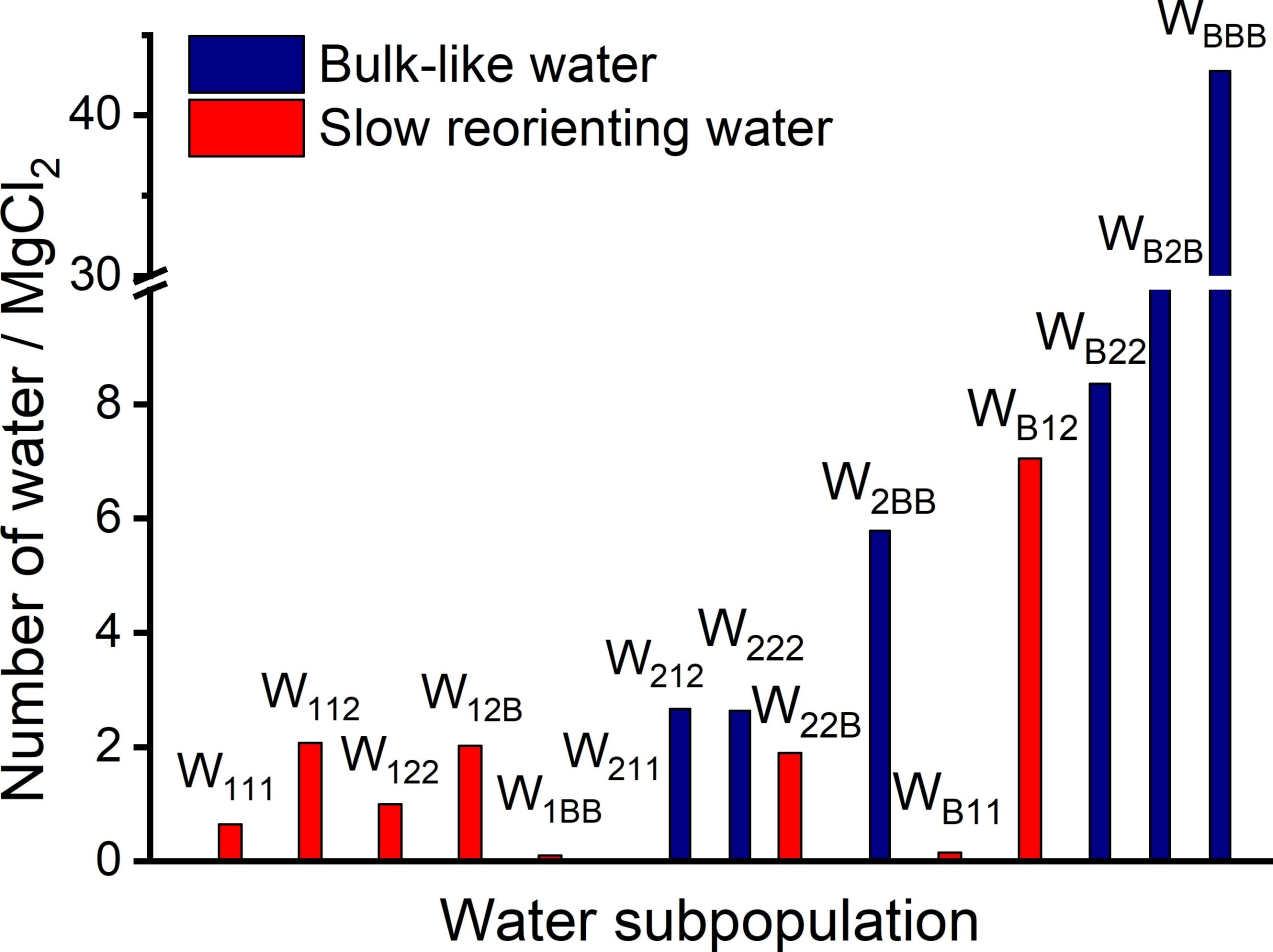
Distribution of water molecules in the water subpopulations labelled as bulk‐like (blue) and slow (red) reorienting water dipoles for the 0.6 mol kg^−1^ MgCl_2_ solution. Number of water molecules per MgCl_2_ units. Slow water molecules compared to bulk water molecules are in 6 from the first shell of Mg plus 9 (beyond shell), corresponding to *h*=15 per MgCl_2_ units.

The number of bulk‐like and slow‐orienting molecules in the water subpopulations of the 0.6 mol kg^−1^ MgCl_2_ solution are reported in Figure 10. Here, we define the hydration number (*h*) as the number of water molecules per dissolved MgCl_2_ units that no longer participate in bulk‐like reorientation dynamics. This definition yields an *h* value of 15 for the 0.6 mol kg^−1^ MgCl_2_ solution, which corresponds to approximately 6 slow water molecules in the first shell of Mg^2+^ and 9 slow water molecules that are beyond the first shell of this ion. The hydration numbers identified using our computational procedure is independent from the structural definition of the hydration shells and corresponds slow water molecules within or outside the first hydration shells of magnesium and chloride ions.

#### 2.4.4 Concentration‐Dependent Dielectric Spectroscopy of MgCl_2_ Solutions

**Figure 11:**
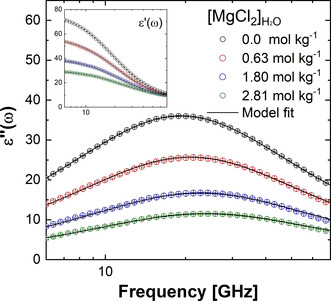
Concentration‐dependent dielectric loss spectrum of aqueous MgCl_2_ solutions. Imaginary and real (inset) components of the double‐Debye dielectric relaxation model.

The concentration‐dependent dielectric loss spectra of MgCl_2_ solutions with concentrations ranging from 0.0 to 2.6 mol kg^−1^ (Figure 11) were analysed by simultaneously fitting the real and imaginary parts of the spectra to the double‐Debye dielectric relaxation model for the frequency dependent dielectric permittivity (*ϵ*):[^50^, ^97^]

(7) $$\epsilon \left(\nu \right)=\frac{S_{1}}{1+i\omega \tau_{1}}+\frac{S_{2}}{1+i\omega \tau_{2}}+\epsilon_{\infty }$$

where *ϵ*_∞_ is the high‐frequency permittivity in aqueous solution, which was set equal to 3.52 based on recent THz spectroscopy experiments of aqueous salt solutions.^[98]^ The first and second terms in Eq. 7 are due to the reorientation motion of water with mode strength *S*_1_ and relaxation time *τ*_1_ (centred at 20 GHz), and mode strength *S*_2_ and relaxation time *τ*_2_ (centred at 1 THz). The conductivity term due to Ohmic loss from the ion conductivity was removed by conducting independent conductivity experiments, such that only dielectric relaxation contribution from dipole fluctuation was considered in the fitting process. According to the extended Cavell equation,^[99]^ the intensities of the relaxation modes *S*_1_ and *S*_2_ are proportional to the number of molecules participating to each mode. The dielectric strength of an aqueous electrolyte solution with concentration *c*, *S*(*c*)=*S*_1_(*c*)+*S*_2_(*c*), is generally less than that of neat water due to the following depolarization effects:^[88]^ dilution effect, ions in solutions reduce the number of waters per unit volume resulting in the decrease of dipole concentration; kinetic depolarization, under the influence of an external electric field, ions in solution diffuse according to the direction of the field, inducing a reorientation of the surrounding water molecules that is opposite to the direction of the field; static depolarization, some water molecules in the solution are bound or strongly affected by the presence of ions and are oriented towards the local ionic field caused. These water molecules have a slow reorientation dynamics with a relaxation time that is outside the GHz‐to‐THz window in which the reorientation process of bulk‐like water occurs.^[87]^ Therefore, the water molecules in an electrolyte solution which reorientation dynamics is retarded with respect to bulk behaviour do not contribute to the GHz‐to‐THz dielectric spectrum, leading to an additional dielectric loss in this frequency range. In DRS, the hydration number of an aqueous electrolyte solution is defined as the number of water molecules characterized by a slow reorientation dynamic and that do not contribute to the bulk‐like relaxation process. To determine the hydration numbers of MgCl_2_ solutions from the dielectric loss spectra, first the static depolarization component is computed, according to the total depolarization model, as the difference between the total dielectric loss (▵S_total_) and the kinetic depolarization contribution (▵S_kinetic_):[^49^, ^97^]

(8) $$\Delta S_{static}=\Delta S_{total}-\Delta S_{kinetic}=\Delta S\left(c\right)-\sigma \left(c\right)\cdot \frac{2\tau_{1}\left(0\right)}{3}\frac{\epsilon_{\infty }\left(c\right)-\epsilon_{s}\left(0\right)}{\epsilon_{s}\left(0\right)\epsilon_{0}}$$

where *ϵ*_s_(0) is the static permittivity of pure water, *ϵ*_∞_(*c*) is the high‐frequency permittivity of the aqueous electrolyte solution, and σ(*c*) is the conductivity of electrolyte solution with concentration *c*. The expression for the kinetic depolarization assumes that water dipoles rotate to the opposite direction of the external field and according to their reorientation time scale. Therefore, *τ*_1_(0) is the reorientation time of the bulk water relaxation mode, and the 2/3 factor originates from the assumption that the flow of solvent at the ion surface is governed by the perfect slip boundary condition.^[97]^ By considering the dilution effect, the number of slow water molecules (hydration number) is obtained using the following expression:

(9) $$N_{hyd}=\frac{\left(c_{H_{2}O}\left(c\right)-\frac{S\left(0\right)+\Delta S_{static}}{S\left(0\right)}c_{H_{2}O}\left(0\right)\right)}{c}$$

where *c*_H2O_(*c*) and *c* represents the concentration of water and of solute, respectively. In THz‐DR experiments, larger amounts of depolarization were measured than depolarization by dilution effect and kinetic depolarization. This amount of depolarization can be explained by the presence of the water molecules which no longer participate in bulk‐like reorientation dynamics. This corresponds to the hydration water.[^87^, ^88^]

#### 2.4.5 Concentration Dependence of the Hydration Number

**Figure 12:**
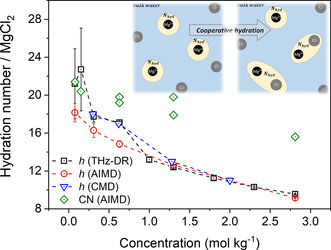
The hydration numbers (*h*) of MgCl_2_ as a function of concentration computed from the water reorientation dynamic analysis of *ab initio* and classical MD simulations, and from the THz‐DR experiments. Standard errors of the MD values obtained from the variations of block averages, each lasting 5 ps, using four different time origins. Also reported are values of the integration of the Mg−O radial distribution functions at the second minima, which gives the coordination number (CN) of the first and second shell.

Figure 12 compares the values of *h* for aqueous MgCl_2_ solutions with concentrations ranging from 0.1 to 2.8 mol kg^−1^ obtained computationally from the *ab initio* MD simulations and experimentally from the concentration‐dependent dielectric loss spectra. The connection between single‐water molecules reorientational dynamics and the values obtained from DRS measurements is discussed in Supporting Information. The theoretical values are in good agreement with the experimental hydration numbers, especially at higher concentrations. The two **h(c)** profiles diminish as the concentration increases because of the cooperative hydration of Mg^2+^ and Cl^−^ ions beyond their first hydration shells (Figure 12, *inset*), which corresponds to a decrease of the number of water molecules belonging to the first and second shells of Mg^2+^ with an increase in salt concentration. Since *ab initio* MD simulations is a very computational intensive technique, it would be more advantageous using trajectories generated from classical MD. Divalent cations such as Mg^2+^ represent a challenge for empirical forcefield calculations, particularly regarding the treatment of overpolarization and Coulombic singularities,^[100]^ with several works discussing the importance of polarization and charge transfer in modulating the properties of water.[^101^, ^102^] A simple approach to improve force fields for electrolyte solutions is the electronic continuum correction (ECC).[^100^, ^103^, ^104^] In this approach, the fast electronic polarization is taken into account in a mean field approach and implemented numerically by scaling the charges of the ions. Here, we have used the Lennard‐Jones potentials for MgCl_2_ solutions parameterized by Duboue‐Dijon et al. named ECC,^[45]^ in which the charges for the magnesium and chlorine ions are set to +1.7 and −0.85, respectively, and the water molecules are represented by SPC/E. Di Tommaso and co‐workers have conducted a detailed assessment of several nonpolarizable interatomic potentials for hydrated Mg^2+^ by computing the energetic (gas phase dissociation reaction of Mg(H_2_O)_6_), structural (Mg‐water radial and angular distribution functions), dynamic (velocity‐autocorrelation function of Mg), and kinetic (free energy of Mg‐dehydration) properties.^[46]^ Overall, the potential model of Duboue‐Dijon (ECC big) provided the best agreement with respect to quantum mechanical and experimental reference data. The retardation factor associated with the water subpopulations of MgCl_2_ was determined with the approach used for *ab initio* MD (Figure S15 in Supporting Information), from which the hydration numbers of MgCl_2_(aq) was computed as a function of the salt concentration. Figure 12 shows the hydration numbers generated from the classical MD trajectories of MgCl_2_ (100 ps) are consistent with the *ab initio* MD result and in good agreement with THz‐DR experiments, especially at lower concentration, probably because of the longer simulation period. Figure 12 also reports the average coordination number (CN) of the first and second shell of Mg^2+^ as a function of the MgCl_2_ concentration, which were obtained from the integration of the Mg−O radial distribution functions at the second minima. Except for the more dilute solution, the values of CN are too high compared with the hydration numbers obtained from the water reorientation dynamic analysis. Since the coordination number is based on a simple spatial definition, it cannot capture the subtle cooperative hydration effects of Mg^2+^ and Cl^−^ on the dynamical properties of the surrounding water molecules.

## 3 Conclusions

By means of *ab initio* MD simulations, we have investigated the structural and low‐frequency dynamics of aqueous MgCl_2_ solutions, with concentrations ranging from 0.1 to 2.8 mol kg^−1^. Compared with bulk water, MgCl_2_ has a considerable influence on the water‐water hydrogen bonding network, as confirmed by detailed analysis of the radial distribution function, hydrogen bonding statistics, and vibrational density of states, which weh ave ascribed to the specific strong Mg‐water interaction. The dynamics of the hydrogen bond network solutions was characterized in terms of the water‐water hydrogen bonding lifetimes and water dipole reorientation. The presence of MgCl_2_ in solution accelerates the dynamics of hydrogen bond making and breaking because of the weakening of the water‐water interaction strength. These effects have been assigned to the specific strong Mg‐water interaction rather than the Cl‐water interaction.

We propose an approach to determine concentration‐dependent values of the hydration number of MgCl_2_(aq) based on the water dipole reorientation dynamics. In this methodology, a hydration status analysis is devised to quantify the cooperative effect of ions on the reorientation dynamics of different water subpopulations in electrolyte solutions. This hydration model provides a tool to characterize the behavior of water in the hydration shell of ions and quantify the cation‐anion mixture effect on water reorientational dynamics. We have found that water molecules that are in the first shell of Mg^2+^, and water molecules that are in the second shell of Mg^2+^ and the first shell of Cl^−^ have a retarded reorientation dynamics compared with bulk‐like behavior, but no cooperative long‐range effects are observed for water molecules that are in the second hydration shell of Mg^2+^ and Cl^−^. The hydration number is determined from the number of moles of water molecules per mole of dissolved salt that no longer participate in bulk‐like reorientation dynamics. The hydration numbers of MgCl_2_ from this *ab initio* MD analysis are in good agreement with those obtained from THz‐DR experiments. This approach is based on a well‐defined criterion for the definition of the hydration number and provides a link between this macroscopic parameter and the molecular‐level processes responsible for affecting the dynamic properties of the solution compared with bulk‐liquid water.

## Supporting Information

Structural properties of the cation‐water hydration shell. Details of the simulated solutions. Ion pairing of magnesium chloride. Ion‐water radial distribution function analysis of MgCl_2_ solutions. Distribution of hydrogen bonds in water and electrolyte solutions. In‐shell hydrogen bond strength. Hydrogen bond relaxation times. Protocol for the calculation of time correlation functions. Time average of time correlation functions over several time origin. Water subpopulations in MgCl_2_ solutions. Procedure for the categorization of water molecules. Water exchange between different subpopulations. Temporal variation of the average number of H_2_O among different subpopulations. Reorientation time correlation function of hydrated Mg^2+^ and Cl^−^. Reorientation time correlation function analysis from classical MD. Connection between single‐water molecule reorientational dynamics and dielectric relaxation spectroscopy measurements.

## Conflict of interest

The authors declare no conflict of interest.

## Supporting information

As a service to our authors and readers, this journal provides supporting information supplied by the authors. Such materials are peer reviewed and may be re‐organized for online delivery, but are not copy‐edited or typeset. Technical support issues arising from supporting information (other than missing files) should be addressed to the authors.

Supplementary
