# Supplementary material for: Hydrogen‐Bond Structure and Low‐Frequency Dynamics of Electrolyte Solutions: Hydration Numbers from ab Initio Water Reorientation Dynamics and Dielectric Relaxation Spectroscopy
Source: Chemphyschem. 2020 Sep 30;21(20):2334–46. doi: 10.1002/cphc.202000498 (PMC7702081; doi:10.1002/cphc.202000498)
Supplement: Supplementary file 1 — Supplementary [file CPHC-21-2334-s001.pdf]

ChemPhysChem

Supporting Information

Hydrogen-Bond Structure and Low-Frequency Dynamics of Electrolyte Solutions: Hydration Numbers from ab Initio Water Reorientation Dynamics and Dielectric Relaxation Spectroscopy

Seonmyeong Kim, Xiangwen Wang, Jeongmin Jang, Kihoon Eom, Simon L. Clegg, Gun-Sik Park,* and Devis Di Tommaso*

Table of Contents

Details of the simulated solutions 3
Ion pairing of magnesium chloride 4
Ion-water radial distribution function analysis of $MgCl_2$ solutions 5
Distribution of hydrogen bonds in water and electrolyte solutions 6
In-shell hydrogen bond strength 8
Hydrogen bond relaxation times 10
Protocol for the calculation of time correlation functions 11
    Time average of time correlation functions over several time origin 12
Water subpopulations in $MgCl_2$ solutions 15
    Procedure for the categorization of water molecules 15
    Water exchange between different subpopulations 17
    Temporal variation of the average number of H_2O among different subpopulations 19
Reorientation time correlation function of hydrated Mg^{2+} and Cl^{-} 20
Reorientation time correlation function analysis from classical MD 21
Connection between single-water molecule reorientational dynamics and dielectric relaxation spectroscopy measurements 22
References 24

Structural properties of the cation–water hydration shell

TABLE S1. Structural properties of the cation–water radial distribution functions obtained from the *ab initio* MD simulations of the hydrated ions (isolated ion, no counterion). The positions, r_{max}, and amplitudes, $g(r_{max})$, of first peak and the average coordination number (CN) of the cation hydration shell are compared with other *ab initio* MD studies and available experimental data. Distances in Å.

System	Reference	Method a)	Functional	Pseudopot. b)	Basis set c)	r_{max}	$g(r_{max})$	CN
Na^+	This study	BOMD	PBE-D3	GTH	DZVP/PW (1000 Ry)	2.41	5.0	5.1
		BOMD	revPBE-D3	GTH	DZVP/PW (1000 Ry)	2.53	4.6	5.6
	Duignan et al.[1]	BOMD	revPBE-D3	GTH	DZVP/PW (400 Ry)	2.51	4.1	
		BOMD	SCAN	GTH	DZVP/PW (1200 Ry)	2.36	5.9	
	Galib et al.[2]	BOMD	revPBE	GTH	TZV2P/PW (400 Ry)	2.45	5.8	5.7
		BOMD	revPBE-D3	GTH	TZV2P/PW (400 Ry)	2.53	4.8	6.0
		BOMD	revPBE	GTH	DZVP/PW (400 Ry)	2.46	5.7	5.7
		BOMD	revPBE-D3	GTH	DZVP/PW (400 Ry)	2.56	4.0	6.1
		BOMD	BLYP	GTH	TZV2P/PW (400 Ry)	2.40	5.9	4.9
		BOMD	BLYP-D2	GTH	TZV2P/PW (400 Ry)	2.46	6.4	5.7
NaCl (6 m)	Galib et al.[2]	EXAFS				2.37		5.4
		XRD				2.38		5.5
NaCl (2.5 m)	Galib et al.[2]	XRD				2.38		5.9
K^+	This study	BOMD	PBE-D3	GTH	DZVP/PW (1000 Ry)	2.80	3.4	6.2
	Duignan et al.[1]	BOMD	revPBE-D3	GTH	DZVP/PW (400 Ry)	2.98	3.1	
		BOMD	SCAN	GTH	DZVP/PW (1200 Ry)	2.78	3.8	
	Glezakou et al.[3]	EXAFS				2.76		6.1
Cs^+	This study	BOMD	PBE-D3	GTH	DZVP/PW (1000 Ry)	3.17	2.7	5.9
	Roy et al.[4]	BOMD	PBE-D3	PAW	PW (30 Ry)	3.15	3.3	
Mg^{2+}	This study	BOMD	PBE-D3	GTH	DZVP/PW (1000 Ry)	2.11	13.5	6.0
	Di Tommaso[5]	CPMD	PBE	USPP	PW (30 Ry)	2.08	12.3	6.0
	Callahan et al.[6]	XRD				2.0-2.12		6.0
Ca^{2+}	This study	BOMD	PBE	GTH	DZVP/PW (1000 Ry)	2.40	10.8	6.0
		BOMD	PBE-D3	GTH	DZVP/PW (1000 Ry)	2.40	10.2	6.0
		BOMD	BLYP	GTH	DZVP/PW (1000 Ry)	2.40	9.6	6.3
		BOMD	BLYP-D3	GTH	DZVP/PW (1000 Ry)	2.42	9.9	6.8
		BOMD	revPBE	GTH	DZVP/PW (1000 Ry)	2.43	9.4	6.6
		BOMD	revPBE-D3	GTH	DZVP/PW (1000 Ry)	2.39	10.8	6.1
	Di Tommaso et al.[5]	CPMD	PBE-D3	GTH	DZVP/PW (1000 Ry)	2.36	8.5	6.4
	Bako et al.[7]	CPMD	BLYP	NCPP	PW (70 Ry)	2.45	10.5	6
$CaCl_2$ (6 m)	Fulton et al.[8]					2.43		7.2

a) BOMD = Born-Oppenheimer molecular dynamics, CPMD = Car-Parrinello Molecular Dynamics. b) GTH = Goedecker-Teter-Hutter; USPP = Ultra-Soft Pseudopotential; PAW = projector augmented wave; NCPP = Norm-Conserving Pseudopotential. c) DZVP = double-zeta valence polarized; TZV2P = triple-zeta valence doubly polarized; PW = plane wave.

Details of the simulated solutions

TABLE S2. Details of the electrolyte solutions: concentration (*c*) in mol.kg^{-1}, number of units and H_2O molecules, cell length after classical MD (NPT) simulation, presence of Mg^{2+}–Cl^- contact ion pairs at the initial coordinates of the ab initio MD simulation of $MgCl_2$ solutions.

System		*c* (mol.kg^{-1})	n_{pairs}	n_{water}	Cell length (Å)	Mg–Cl ion pairing
Bulk water	1	0.00	0	729	28.0	
$MgCl_2$	2	0.08	1	726	28.0	*without* ion pairing
	3	0.15	2	723	27.8	*without* ion pairing
	4	0.63	8	705	27.8	*without* ion pairing
	5	0.63	8	705	27.7	*with* ion pairing
	6	1.30	16	681	27.7	*without* ion pairing
	7	1.30	16	681	27.7	*with* ion pairing
	8	2.81	32	633	27.4	*with* ion pairing
CsCl	9	0.63	16	710	27.7	
Mg^{2+}	10	0.88	1	63	12.3	
Ca^{2+}	11	0.88	1	63	12.4	
K^+	12	0.88	1	63	12.5	
Na^+	13	0.88	1	63	12.5	
Cs^+	14	0.88	1	63	12.5	
Cl^-	15	0.88	1	63	12.5	

Ion pairing of magnesium chloride

TABLE S3. The speciation of magnesium chloride ion pairs as a function of concentration determined in terms of the following ion pairing criteria: contact ion pair (CIP) when Mg^{2+} and Cl^{-} are in direct physical contact; a SSHIP is considered when Mg^{2+} and Cl^{-} are separated by one water molecule; solvent-shared ion pairs (SSHIP) when Mg^{2+} and Cl^{-} are separated by one water molecule; solvent-separated ion pairs (SSIP) when Mg^{2+} and Cl^{-} are separated by two water molecules; free ion pairs (FIP) when the above conditions are not met, and the cation and anion are fully hydrated beyond the second hydration shell. Assignments made according to the analysis of the Mg–O and Mg–Cl radial distribution functions (RDFs) in Figure S3.1: CIP if $r_{Mg-Cl} \leq r_{Mg-Cl}^{min1}$; SSHIP if $r_{Mg-Cl}^{min1} < r_{Mg-Cl} \leq r_{Mg-Cl}^{min2}$; SSIP if $r_{Mg-Cl}^{min2} < r_{Mg-Cl} \leq \left(r_{Mg-O}^{min2} + r_{Cl-O}^{min2}\right)$, where r_{Mg-Cl}^{min1} and r_{Mg-Cl}^{min2} are the positions of the first and second minima, respectively, of the Mg–Cl, Mg–O and Cl–O radial distribution functions (Figure S1), where r_{Mg-X}^{min1}, r_{Mg-X}^{min2} are the positions of the first and second minima, respectively, of the M–X (X = O and Cl) radial distribution function, and r_{Cl-O}^{min2} is the position of the second minima of the Cl–O RDF.

System	c (mol.kg^{-1})	CIP (%)	SSHIP (%)	SSIP (%)
2	0.08	0.0	29.8	70.2
3	0.15	0.0	0.0	100.0
4	0.63	0.0	58.4	35.0
5	0.63	25.0	32.1	42.9
6	1.30	0.0	93.4	6.6
7	1.30	50.0	41.9	8.1
8	2.81	56.2	43.7	0.0

Ion-water radial distribution function analysis of $MgCl_2$ solutions

Table S4. Structural properties of the magnesium–water radial distribution functions obtained from the *ab initio* MD simulations of $MgCl_2$ solutions. The positions (r^{max}) and amplitudes [$g(r^{max})$] of first and second peaks, the average coordination number of the first (CN_1) and second (CN_2) hydration shells are compared with available experimental data. Distances in Angstrom.

System	Concentration	r_1^{max}	$g(r_1^{max})$	r_2^{max}	$g(r_2^{max})$	CN_1	CN_2
2	0.08	2.11	14.7	4.35	2.6	6.0	15.4
3	0.15	2.11	14.0	4.14	2.6	6.0	14.4
4	0.63	2.10	15.3	4.23	2.4	6.0	13.8
5	0.63	2.12	14.3	4.21	2.4	5.8	13.4
6	1.30	2.10	15.6	4.20	2.4	6.0	13.8
7	1.30	2.12	13.5	4.27	2.2	5.4	12.5
8	2.81	2.12	13.4	4.30	1.9	5.1	10.5
Experiment		2.09 ± 0.04		4.1–4.2		6.0	12.0

Distribution of hydrogen bonds in water and electrolyte solutions

TABLE S5. The fraction (*f*) of water molecules with *n* number of hydrogen bonds per water molecule, the average number of hydrogen bonds (n_{HB}) per water molecule in bulk water and in aqueous $MgCl_2$ solutions at different concentrations (mol.kg^{-1}), obtained from *ab initio* MD simulations (PBE-D3). Analysis was based on the following configurational criteria: two water molecules are hydrogen-bonded only if their inter-oxygen distance is less than 3.5 Å and, simultaneously, the hydrogen-oxygen distance is less than 2.45 Å and the oxygen-oxygen–hydrogen angle is less than 30°.[9]

	**System**	***c* (mol.kg^{-1})**	f_0	f_1	f_2	f_3	f_4	f_5	n_{HB}	**Mg^{2+}–Cl^- ion pairing**
Water	1	0.00	0.0	0.6	5.8	21.9	66.8	4.8	3.69	
$MgCl_2$	2	0.08	0.0	0.6	6.4	23.0	65.9	4.0	3.66	*without* ion pairing
	3	0.15	0.0	0.9	8.3	25.7	60.6	4.4	3.59	*without* ion pairing
	4	0.63	0.1	2.0	11.2	27.6	55.4	3.7	3.47	*without* ion pairing
	5	0.63	0.1	1.9	11.3	27.4	56.0	3.3	3.48	*with* ion pairing
	6	1.30	0.4	5.7	29.2	45.2	2.6	0.0	3.21	*without* ion pairing
	7	1.30	0.1	3.7	15.7	32.1	46.0	2.4	3.28	*with* ion pairing
	8	2.81	1.6	11.2	24.7	32.9	28.0	1.5	2.79	*with* ion pairing
CsCl	9	0.63	1.0	6.6	23.6	64.4	4.1	0.0	3.64	

TABLE S6. The fraction (*f*) of water molecules with *n* number of hydrogen bonds per water molecule, the average number of hydrogen bonds (n_{HB}) per water molecule in bulk water and in aqueous $MgCl_2$ solutions at different concentrations (mol.kg^{-1}), obtained from classical MD simulations using the Lennard-Jones potentials developed by Aqvist [10] and Duboue-Dijon et al.[11] to describe the ion-ion and ion-water interactions together with the SPC/E water model. Analysis was based on the following configurational criteria: two water molecules are hydrogen-bonded only if their inter-oxygen distance is less than 3.5 Å and, simultaneously, the hydrogen-oxygen distance is less than 2.45 Å and the oxygen-oxygen–hydrogen angle is less than 30°.[9]

	**Forcefield**	***c* (mol.kg^{-1})**	f_0	f_1	f_2	f_3	f_4	f_5	n_{HB}
Water	SPC/E	0.00	0.0	1.0	8.9	33.2	51.0	5.8	3.52
$MgCl_2$	Aqvist, SPC/E	0.08							
		0.15	0.0	1.4	11.1	33.6	48.3	5.4	3.45
		0.63	0.1	3.1	16.8	34.3	41.2	4.4	3.27
		1.30	0.5	6.1	22.5	34.0	33.4	3.4	3.04
		2.81	2.6	14.9	30.1	20.2	1.8	0.0	2.56
$MgCl_2$	Duboue-Dijon, SPC/E	0.08	0.1	1.2	9.7	32.7	50.5	5.8	3.50
		0.31	0.1	2.0	12.6	33.6	46.6	5.0	3.40
		0.63	0.2	3.2	15.9	33.9	42.2	4.5	3.28
		1.30	0.7	6.8	21.6	33.7	3.5	0.0	3.03
		2.81	3.3	17.2	30.2	29.6	18.1	1.6	2.47
		3.65	6.8	23.5	31.6	25.2	12.0	1.0	2.15

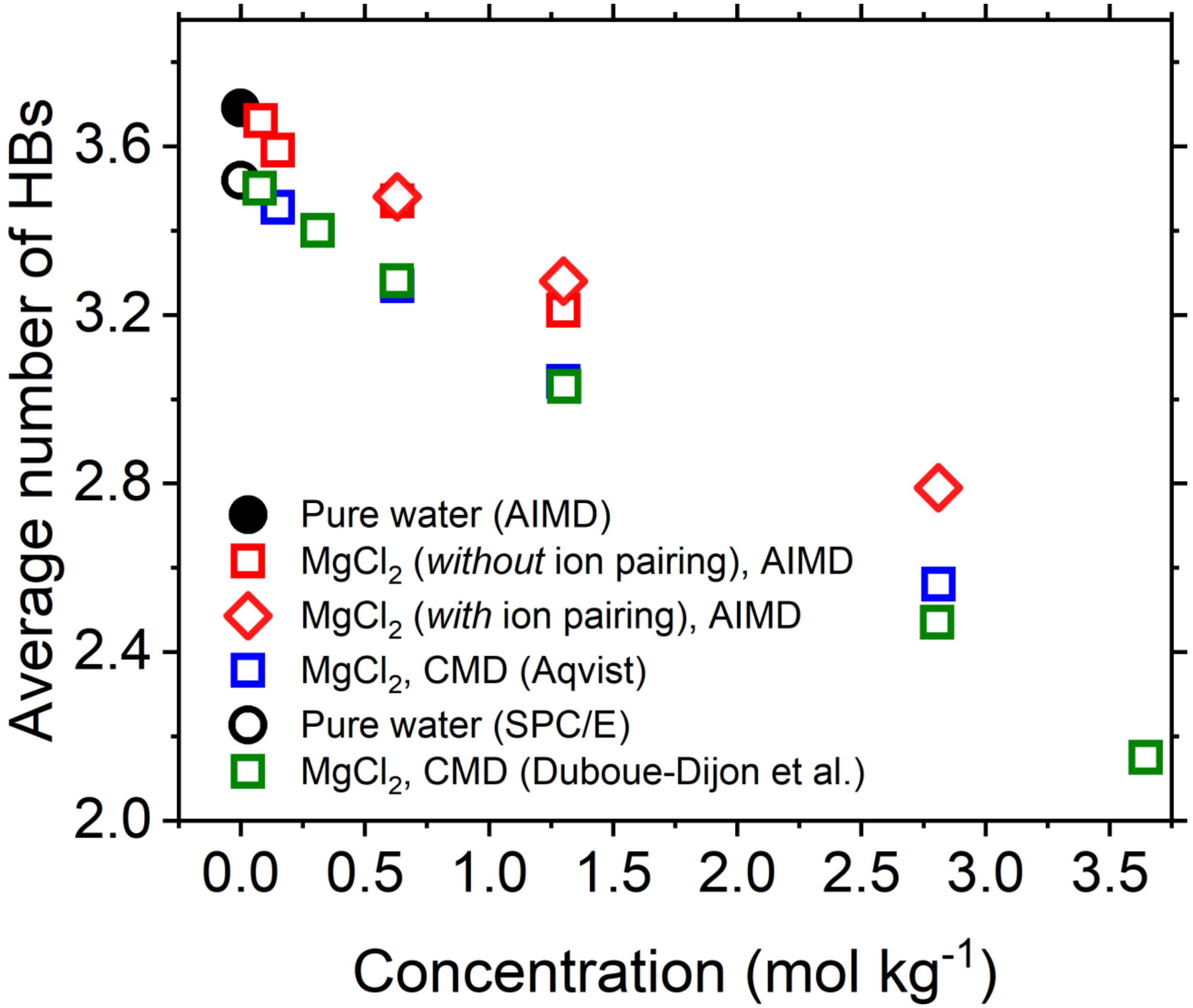

FIGURE S3. Comparison of the average number of hydrogen bonds (HBs) in pure liquid water and aqueous MgCl2 solutions obtained from *ab initio* MD (PBE-D3) and classical MD (Aqvist [10] and Duboue-Dijon et al.[11] forcefields) simulations.

In-shell hydrogen bond strength

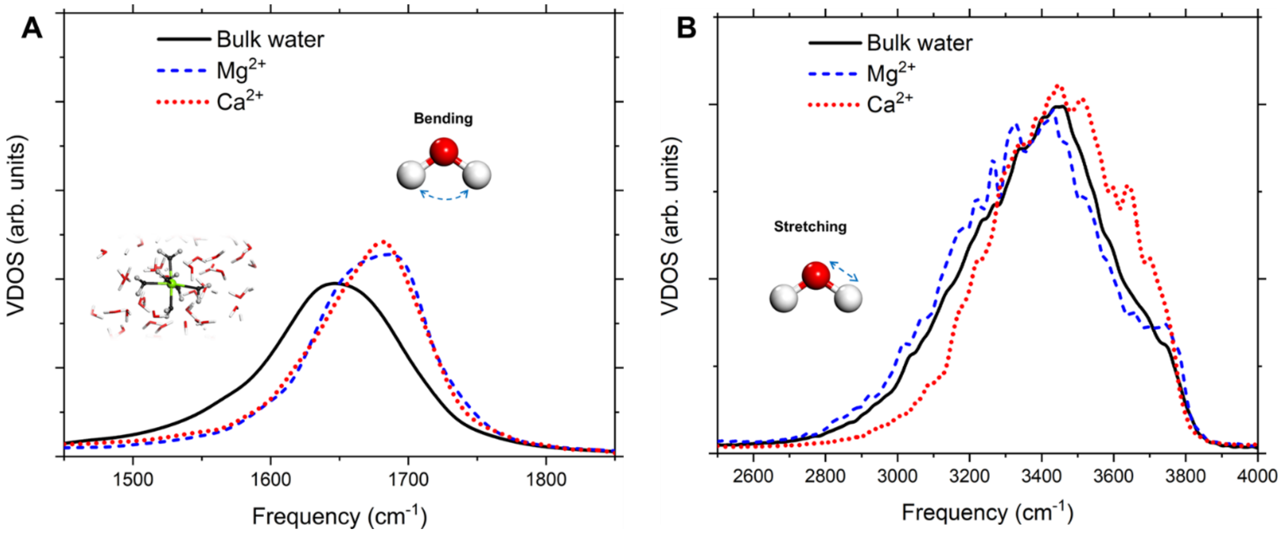

FIGURE S2. Bending (**A**) and stretching (**B**) regions of the vibrational density of states (VDOS) of water. Comparison of pure water and of the water molecules in the first hydration shell of Mg^{2+} and Ca^{2+}.

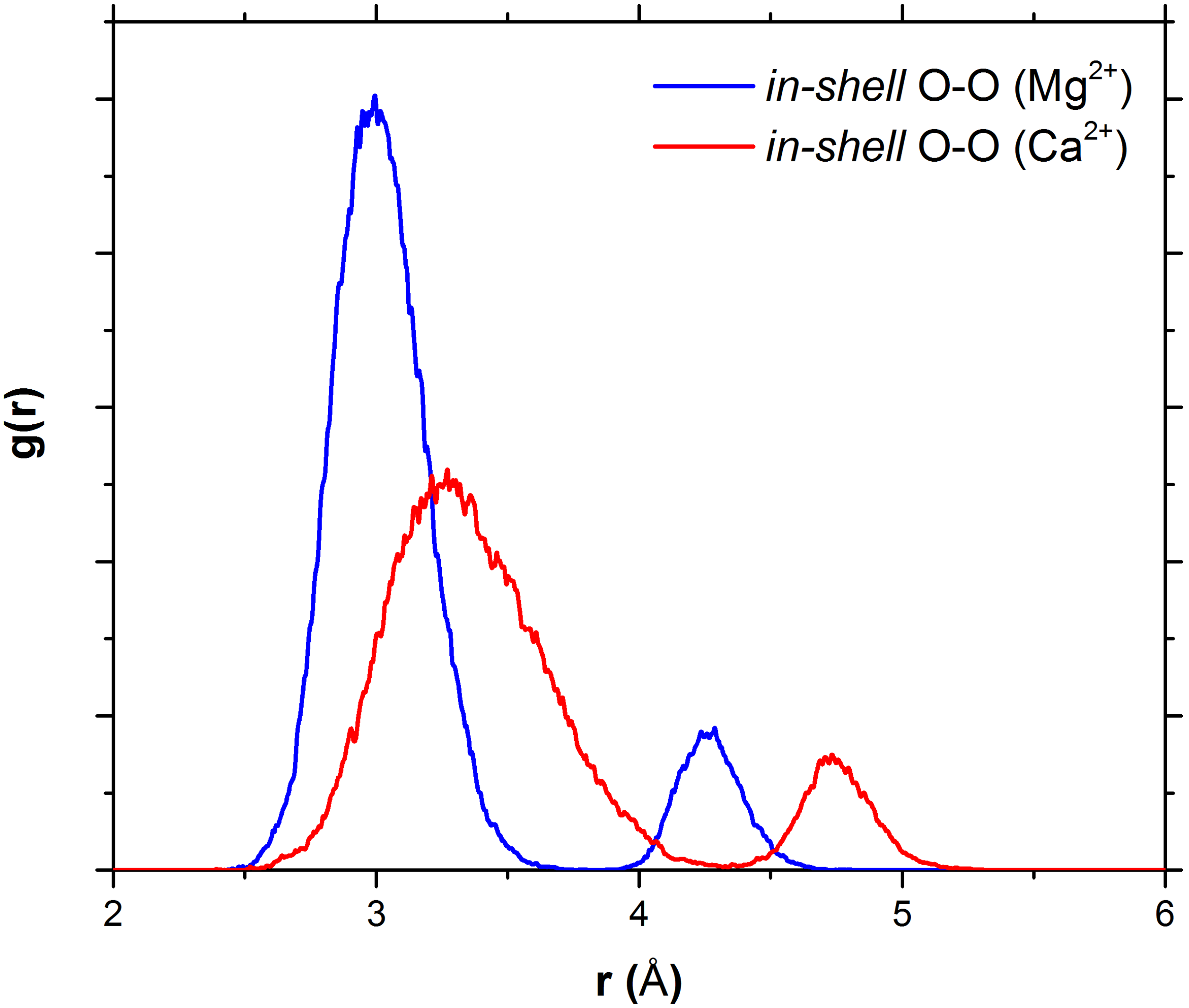

FIGURE S3. Oxygen–oxygen radial distribution functions [g(*r*)] for the water molecules that are part of the hydration shell of Mg^{2+} and Ca^{2+} obtained from *ab initio* MD simulations of isolated ions in solution. For Mg^{2+}, the first peak is shifted to lower distances and is more intense than Ca^{2+}.

Hydrogen bond relaxation times

TABLE S7. Relaxation time, τ_{HB}, computed from the integration of the continuous hydrogen bond correlation function. Relaxation times are expressed in units of ps.

**System**	**c (mol.kg^{-1})**		τ_{HB}
729 H_2O	0.00		1.56
64 H_2O	0.00		1.31
$MgCl_2$ (aq)	0.63	*without* CIP	1.26
	0.63	*with* CIP	1.13
	1.30	*without* CIP	1.22
	1.30	*with* CIP	1.17
	2.81	*with* CIP	1.06
Mg^{2+} (aq)		1st shell	2.09
		2nd shell	1.93
		Overall	1.68
Cl^{-} (aq)		1st shell	1.36
		2nd shell	1.25
		Overall	1.28

Protocol for the calculation of time correlation functions

The methodology below been adopted to compute the hydrogen bonding, , $S_{HB}(t)$, and reorientation time correlation functions (TCF), $P_1(t)$, defined as the first-order Legendre polynomials of water dipole, a unit bisector of the H–O–H angle ($\vec{\mu}$).

1. Calculate dipole vectors of each water molecules at all time step from trajectory, $\mu_i(t)$: dipole vectors of ith water molecule at time t.

2. Compute the inner product between the unit vectors defining the orientation of the dipole moment of the *i*-th water molecule at the time origin t_o and time *t*: $\mathbf{u}_i(t_o) \cdot \mathbf{u}_i(t)$.

3. The first-order Legendre polynomial TCF is obtained from the average over whole water molecule in the simulation box (or in the subpopulation of water molecule):

$$P_1^o(t) = \frac{1}{N_\mathrm{w}} \sum_i^{N_\mathrm{w}} \mathbf{u}_i(t_o) \cdot \mathbf{u}_i(t) \qquad \text{(S1)}$$

4. The first-order Legendre polynomial TCF is obtained for several time origin and overlapping intervals [0, *t*] of equal time length (Figure S3).

5. The value of $P_1(t)$ in the time intervals [0, *t*] is obtain from the average of $P_1^o(t)$:

$$P_1(t) = \frac{1}{N_\mathrm{o}} \sum_i^{N_\mathrm{o}} P_1^o(t) \qquad \text{(S2)}$$

The calculation of the continuous hydrogen bonding time correlation function, $S_{HB}(t)$, adopted a similar protocol.

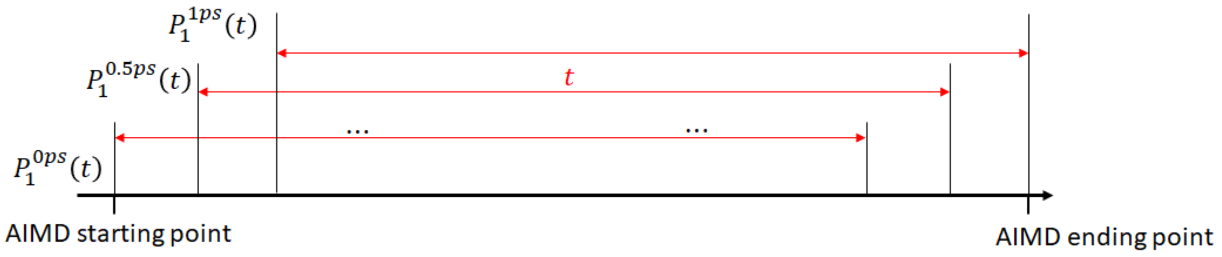

FIGURE S4. Procedure adopted to calculate the value of $P_1^o(t)$ for several time origins and overlapping time of equal time length, [0, *t*] = 16000 fs.

Time average of time correlation functions over several time origin

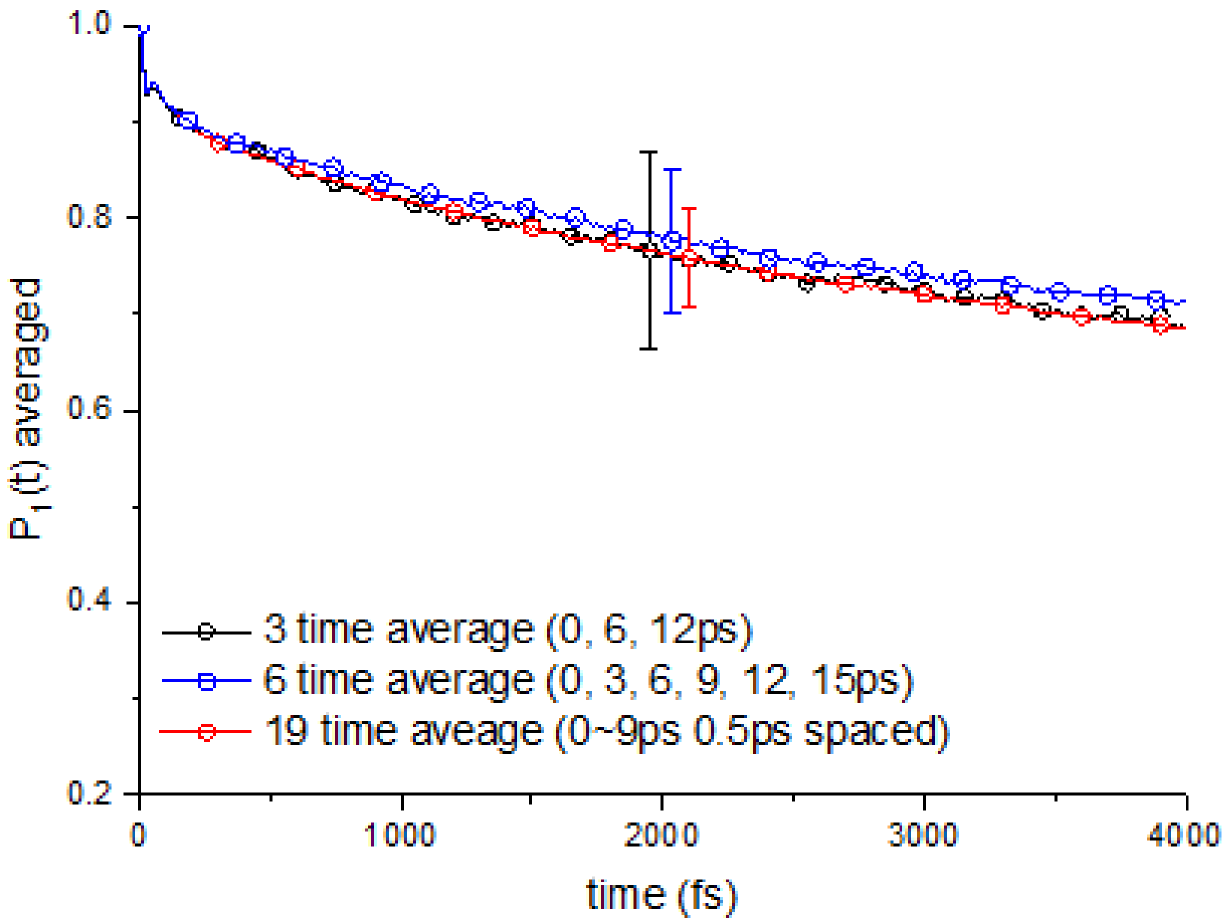

FIGURE S5. First-order Legendre reorientational TCFs, $P_1(t)$, of water in solutions of $MgCl_2$ obtained using indicated time origins. Standard deviation (error bars) decreases by increasing the number of time origins.

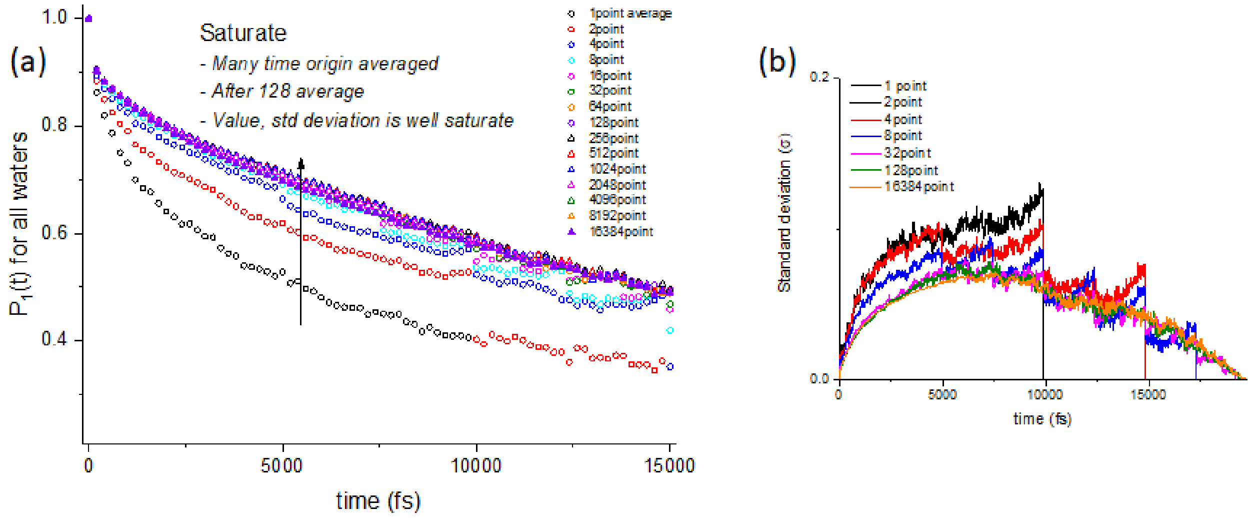

FIGURE S6. First-order Legendre reorientational TCFs, $P_1(t)$, of water in solutions of $MgCl_2$ obtained using indicated time origins. Behavior of the reorientation decay (a) and of the standard deviation (b) with the number of time origins used in the evaluation of $P_1(t)$ (Eq. S2). Time interval used to compute $P_1(t)$ is 16000 steps. Dipole correlation and standard deviation reach convergence after 128 origin time averages.

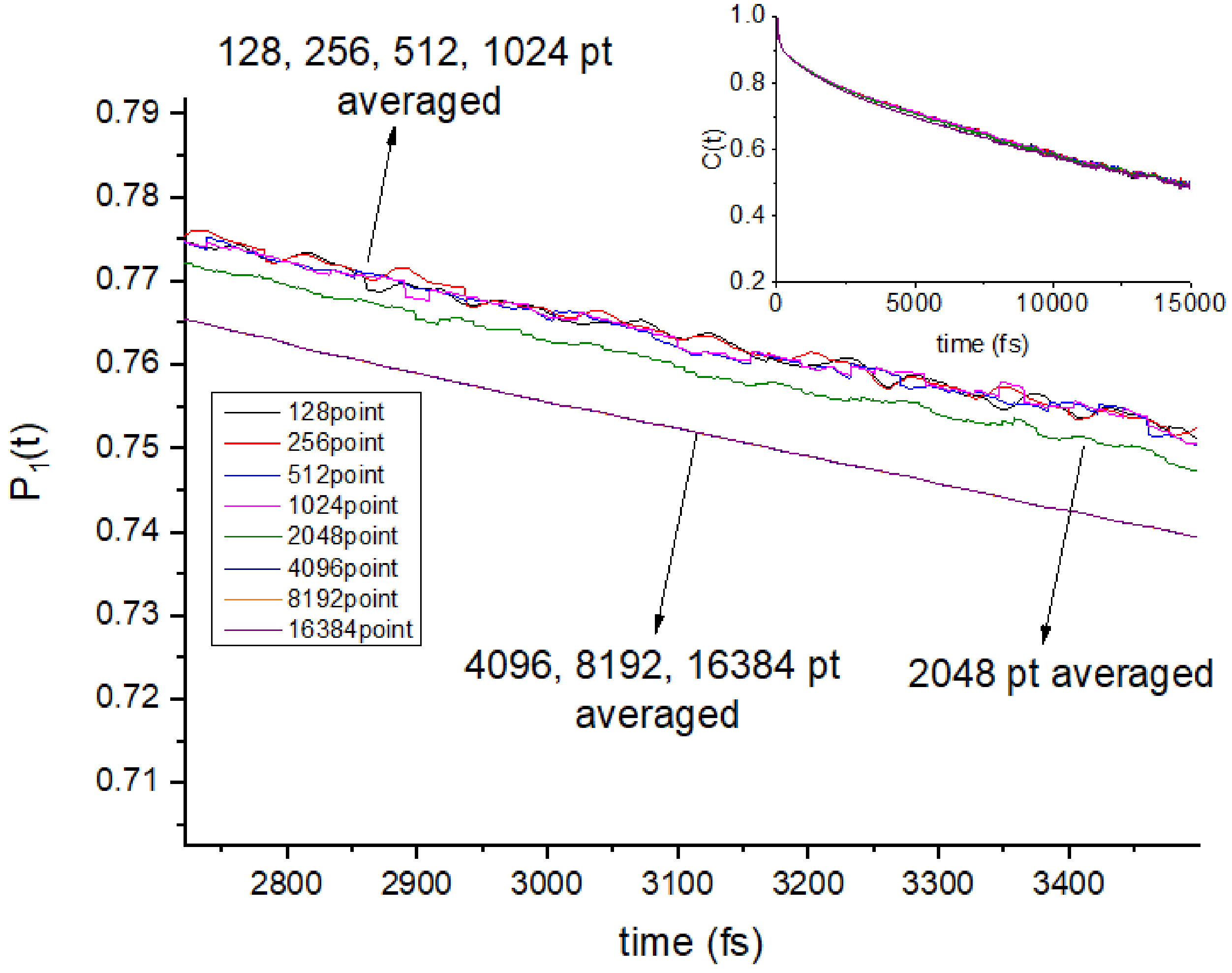

FIGURE S7. First-order Legendre reorientational TCFs, $P_1(t)$, of water in solutions of $MgCl_2$ obtained using indicated time origins. The plots in the time interval [2.8–3.4 ps] show that differences in the computed values of $P_1(t)$ are less than 0.01 (2%).

Water subpopulations in $MgCl_2$ solutions

Procedure for the categorization of water molecules

The hydrogen and oxygen atoms in each water molecule were labelled $O_aH_bH_c$ where *a*, *b*, *c* = 1, 2, and B in terms of the following criteria: 1 when the atom in the first coordination shell of the nearest ion; 2 when the atom is in the second coordination shell of the nearest ion; B when the above conditions are not met. Assignments were made by comparing the distance between O_a and the nearest magnesium ion with position of the first (r_{Mg-O}^{min1}) and second (r_{Mg-O}^{min2}) minima of the Mg–O RDF (Figure S8), and the distance between H_b (or H_c) and the nearest chlorine ion with first (r_{Cl-H}^{min1}) and second (r_{Cl-H}^{min2}) minima of the Cl–H RDF (Figure S9):

i. O_a is labelled O_1 if $r_{Mg-O_1} < r_{Mg-O}^{min1}$;
ii. O_a is labelled O_2 if $r_{Mg-O}^{min1} < r_{Mg-O_1} < r_{Mg-O}^{min2}$;
iii. O_a is labelled O_1 if $r_{Mg-O_1} > r_{Mg-O}^{min2}$; H_b (or H_c) is labelled H_1 if $r_{Cl-H} < r_{Cl-H}^{min1}$;
iv. H_b (or H_c) is labelled H_2 if $r_{Cl-H}^{min1} < r_{Cl-H_b} < r_{Cl-H}^{min2}$;
v. H_b (or H_c) is labelled H_2 if $r_{Cl-H_b} > r_{Cl-H}^{min2}$.

Using these geometrical criteria at time *t* = 0, the water molecules in the $MgCl_2$ solutions were classified in different water subpopulations labelled **Wabc** (Figure S10). For example: **W111** refers to a subpopulation of water molecules of type $O_1H_1H_1$ where oxygen is in the first coordination shell of Mg^{2+} and both hydrogen atoms are in the first coordination shell of Cl^-; **W112** refers to a subpopulation of water molecules of type $O_1H_1H_2$ where oxygen is in the first coordination shell of Mg^{2+}, a hydrogen is in the first coordination shell of Cl^- and the other is the second coordination shell of Cl^-. The continuous HB and dipole reorientational TCFs were then evaluated for all **Wabc** subpopulations.

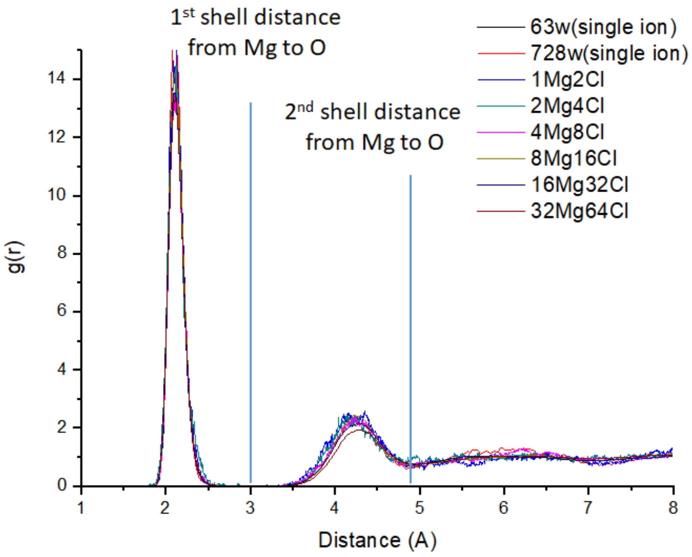

FIGURE S8. Magnesium–oxygen radial distribution functions [g(*r*)] for the $MgCl_2$ solution.

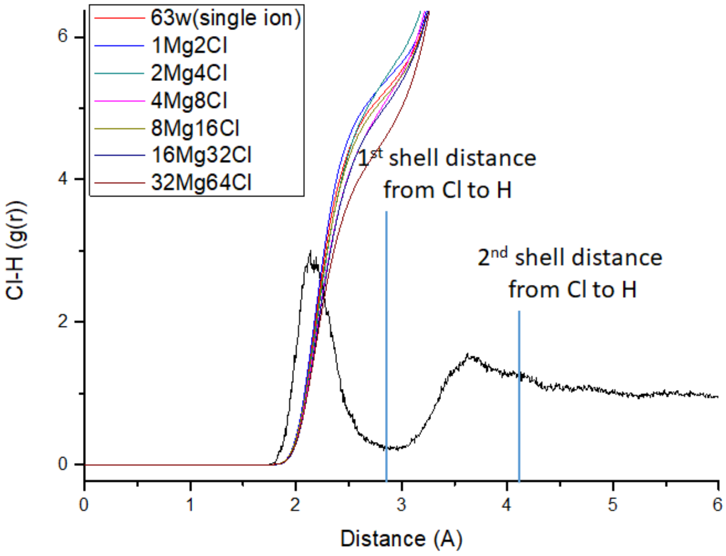

FIGURE S9. Chloride–hydrogen radial distribution functions [g(r)] for the $MgCl_2$ solution.

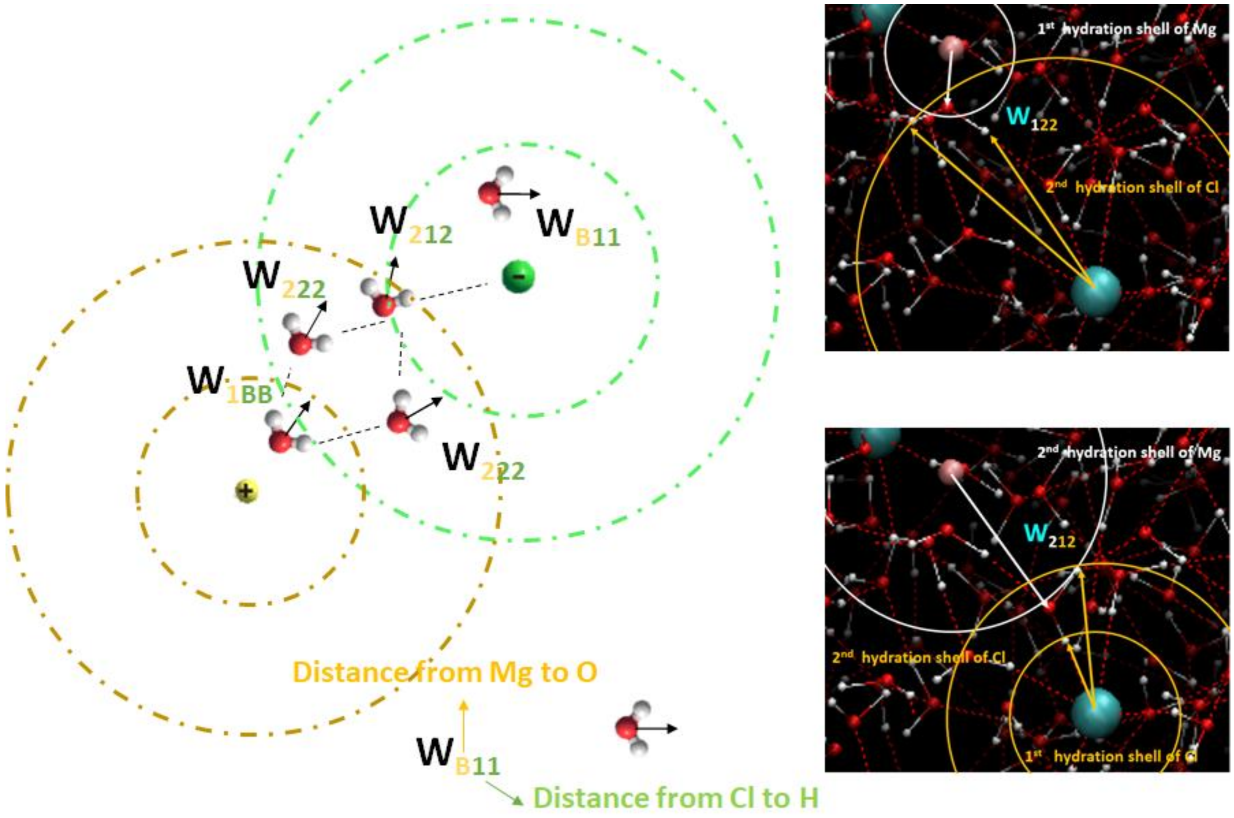

FIGURE S10. Classification of different water molecules into different subpopulation

Water exchange between different subpopulations

In this work, we simply tracked the water molecules belonging to a specific water subpopulation at the time origin (t = 0 ps) for calculating the dipole TCF of each water subpopulation:

$$P_1^o(t) = \frac{1}{N} \sum_{i \in W_{abc}}^{N} \mathrm{u}_i(t_o) \cdot \mathrm{u}_i(t) \qquad \text{(S1)}$$

This approximation has been adopted by other groups to compute time correlation functions of dynamical properties of different water layers at solid/water and air/water interfaces.[12–14] However, water molecules that are in a particular subpopulation at t = 0 ps, e.g. W_{12B} and W_{12B} in Fig. S16, can exchange to "neighbouring" subpopulations. For example, a water molecule that is W_{12B} initially goes to W_{1BB}, a neighbour subpopulation, but molecules in both subpopulations contribute to the hydration water.

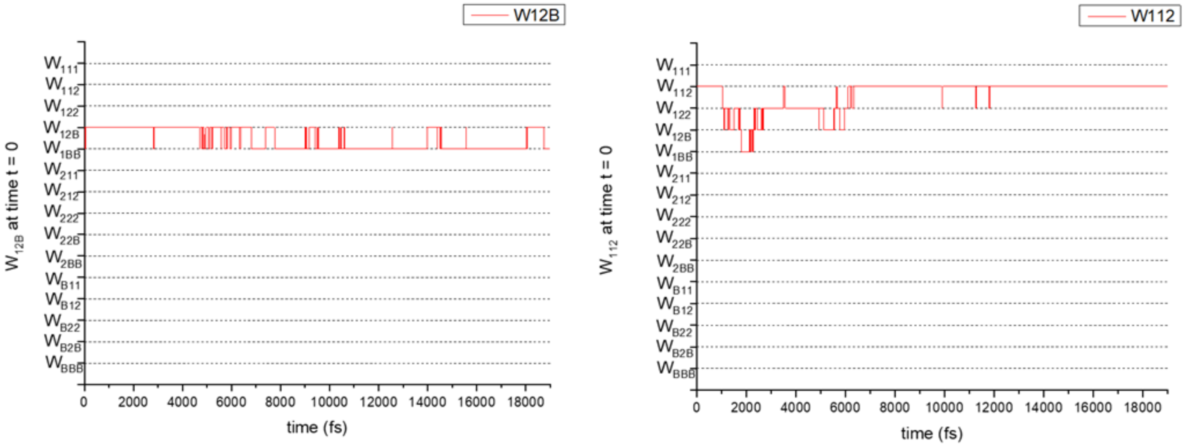

FIGURE S11. Exchange of water molecules between different watr subpopulations. Analysis conducted on the 0.6 mol.kg^{-1} $MgCl_2$ solution.

From the graphs in Fig. S16 we can distinguish two kinds of phenomena: *short-time fluctuations* and *long-term exchange*. Short term fluctuation (left figure) are fast exchanges of a water molecule between the initial subpopulation (t = 0 ps) and neighboring water subpopulations, which can occur because of rotational or vibrational motion of molecules that are at the margin of the distance criteria distinguishing between subpopulations. A long-term exchange (right figure) is seen when a water molecule exists for a considerable time in different water subpopulations and then returns to initial subpopulation or moves into another one; it could occur because of the translational motion of water between neighboring ions. To analyze the long term exchange in more detail, let us consider a simplified hydration model, W_{ab}, that only considers the positions of the oxygen atom: a, b = 1, 2, or B, where 1 corresponds to O in the 1st shell of Mg or Cl, 2 corresponds to O in the 2nd shell of Mg or Cl, and B corresponds to O beyond the 2nd shell of Mg or Cl. Using this simplified classification method, we can exclude the short-term fluctuation and focus on the temporal behavior of the long term exchanges. Let us now define the following function:

$$N_{ab}(t) = \frac{1}{N_{ab}(0)} \sum_{i \in W_{ab}}^{N_{ab}(0)} F_i(t)$$

where $N_{ab}(0)$ corresponds to the number of water molecules in the subpopulation W_{ab} at time t = 0, and the quantity $F_i(t)$ is computed as follows:

$$F_i(t) = \begin{cases} 1 & \text{if } i \in W_{ab} \\ 0 & \text{if } i \notin W_{ab} \end{cases}$$

The normalized, time-dependent function $N_{ab}(t)$ corresponds to the fraction of water molecules initially in W_{ab} that have remained in the same category. Fig. S17 shows that W_{11} (water molecules in the first hydration shell of Mg and Cl) is quite “rigid” as more than 80% of water molecules remained in the original subpopulation. Also, about 80% of water molecule which is initially bulk water is still belongs to W_{BB} under dynamic exchange equilibrium. In case of water molecules in the second shell either Mg^{2+} and Cl^- ion, (W_{B2}, W) between 50 and 60 % remains in the same category. In order to minimize the effect originating from the exchange of water molecules from one subpopulation to another, we used the first 8000 steps data (8ps) of the dipole correlation function to fit the bi-exponential model which give us the averaged relaxation time; this gives more than 70% of water molecules remaining in the initial category. Water molecules of each subpopulation are exchanged with each other, but the relaxation time of dipole correlation function we have obtained is fitted over time intervals that exchange effect is not significant to dipole correlation function because majority of water molecules remain same category.

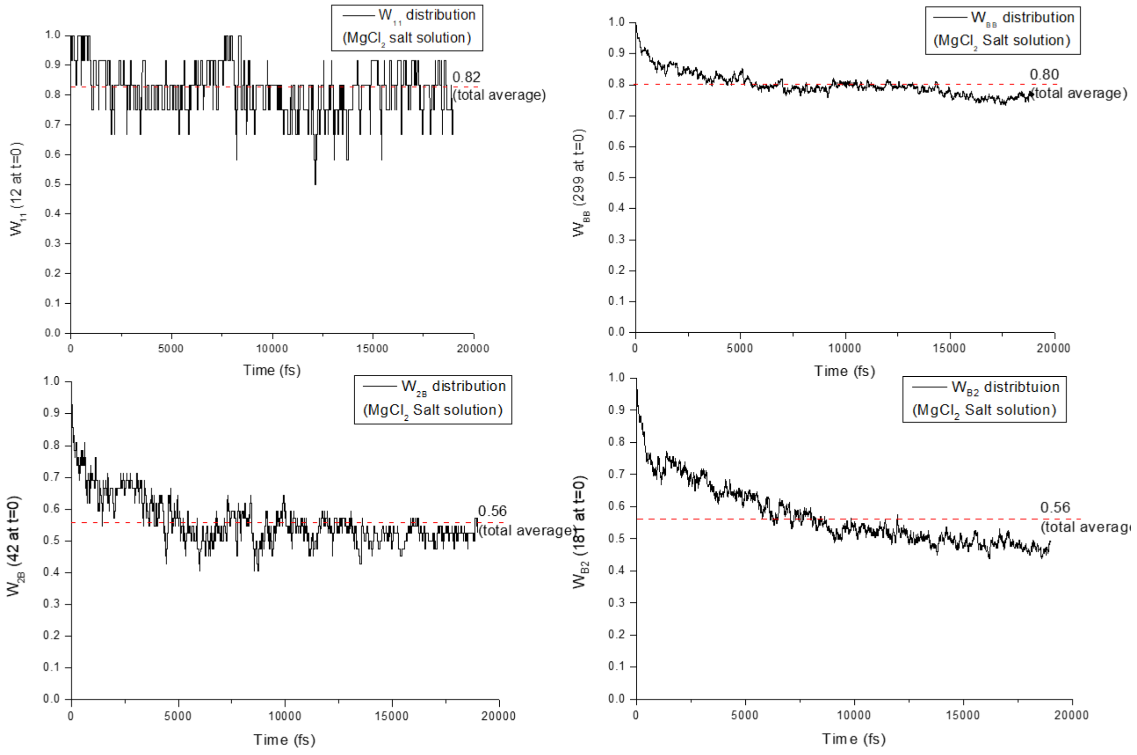

FIGURE S12. Fraction of water molecules that have remained in the same category at time *t*. Analysis of 0.6 mol.kg^{-1} $MgCl_2$ (aq).

Temporal variation of the average number of H_2O among different subpopulations

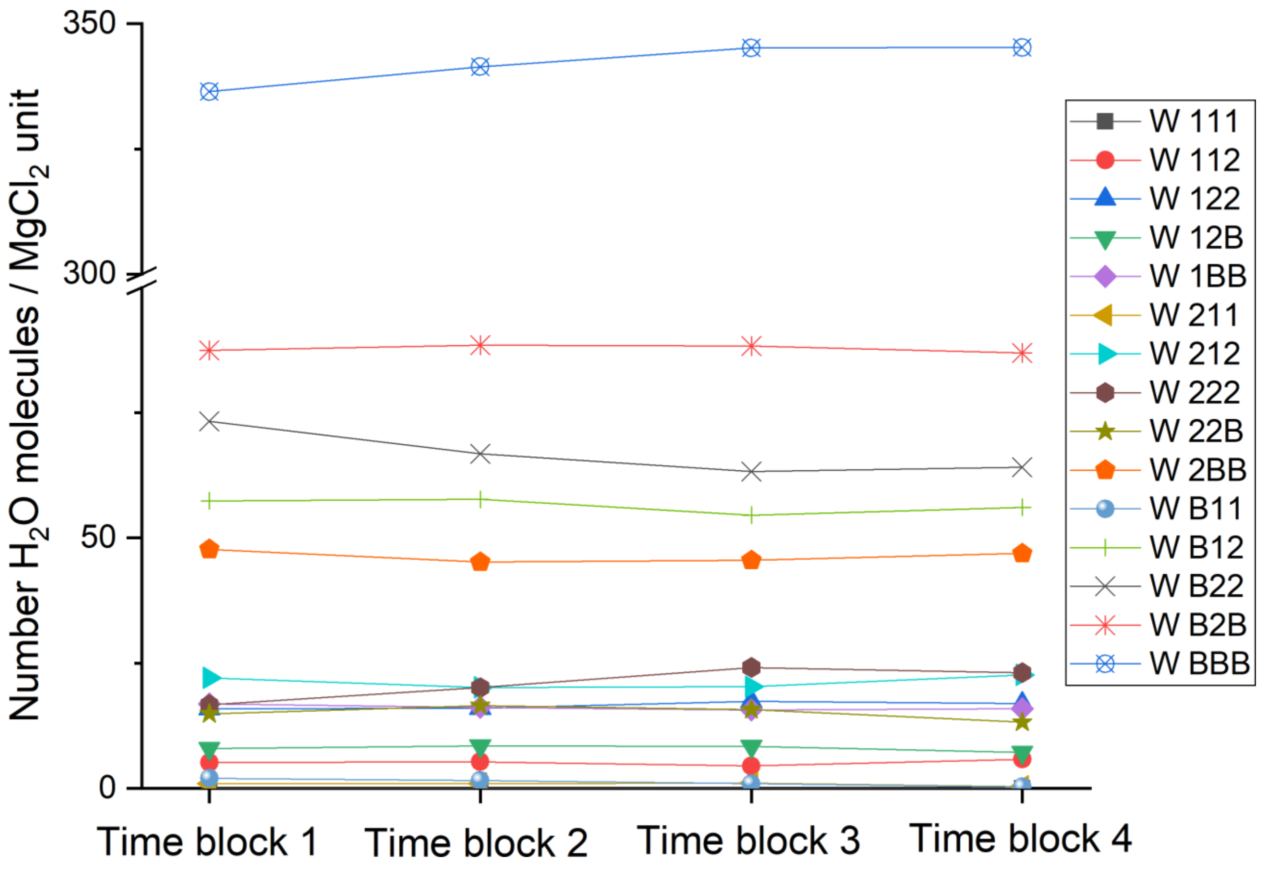

FIGURE S13. Variation of the average number of molecules into the water subpopulations during four consecutive non-overlapping time blocks each lasting 5 ps. The subpopulation analysis was conducted at each time step for the 0.6 mol.kg^{-1} $MgCl_2$ solution.

Reorientation time correlation function of hydrated Mg^{2+} and Cl^-

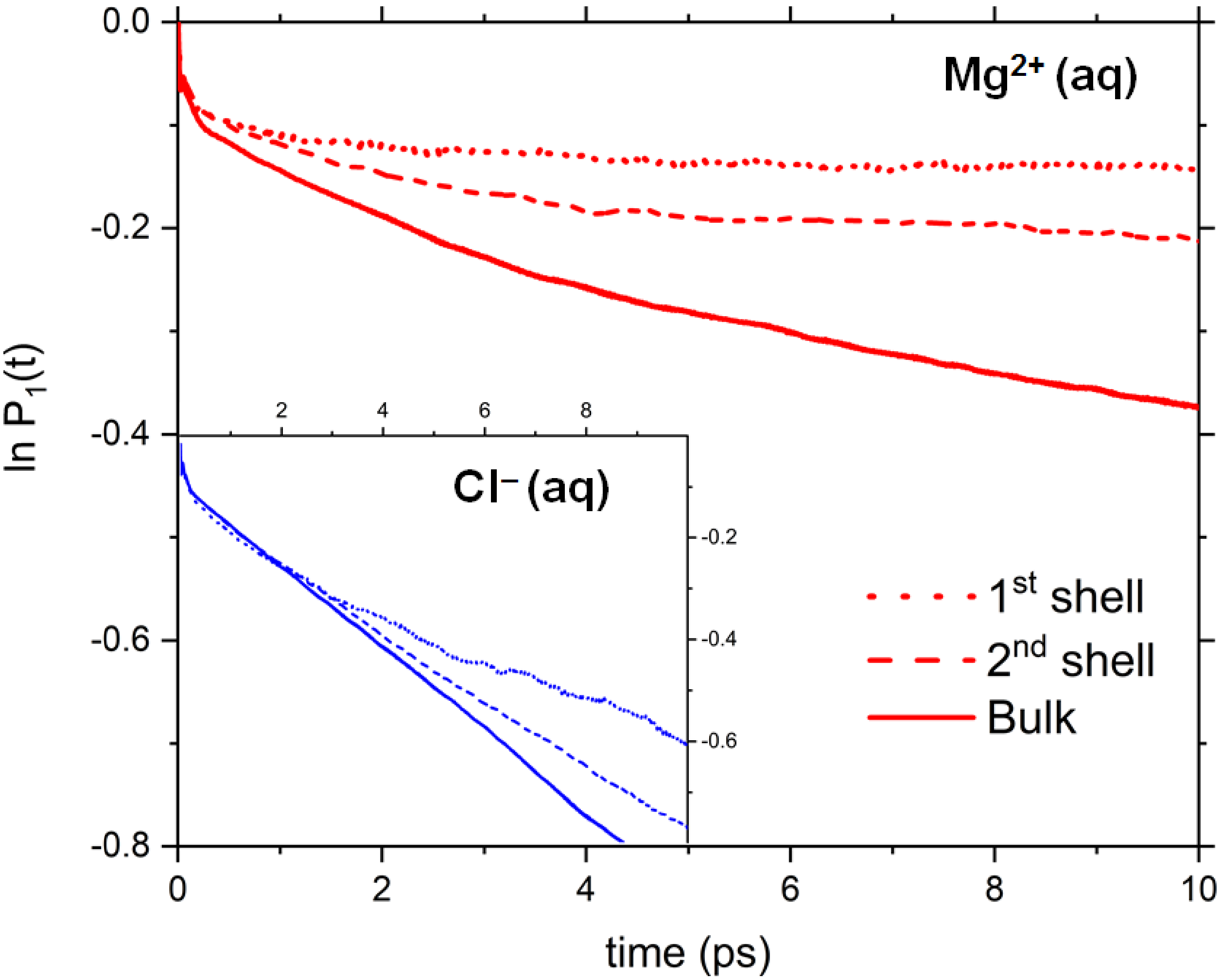

FIGURE S14. First-order Legendre reorientational TCFs, $P_1(t)$, of the water molecules in the first and second coordination shells of Mg^{2+} and Cl^-. Results compared to $P_1(t)$ of the water molecules in the bulk (beyond the second coordination shell of the ion).

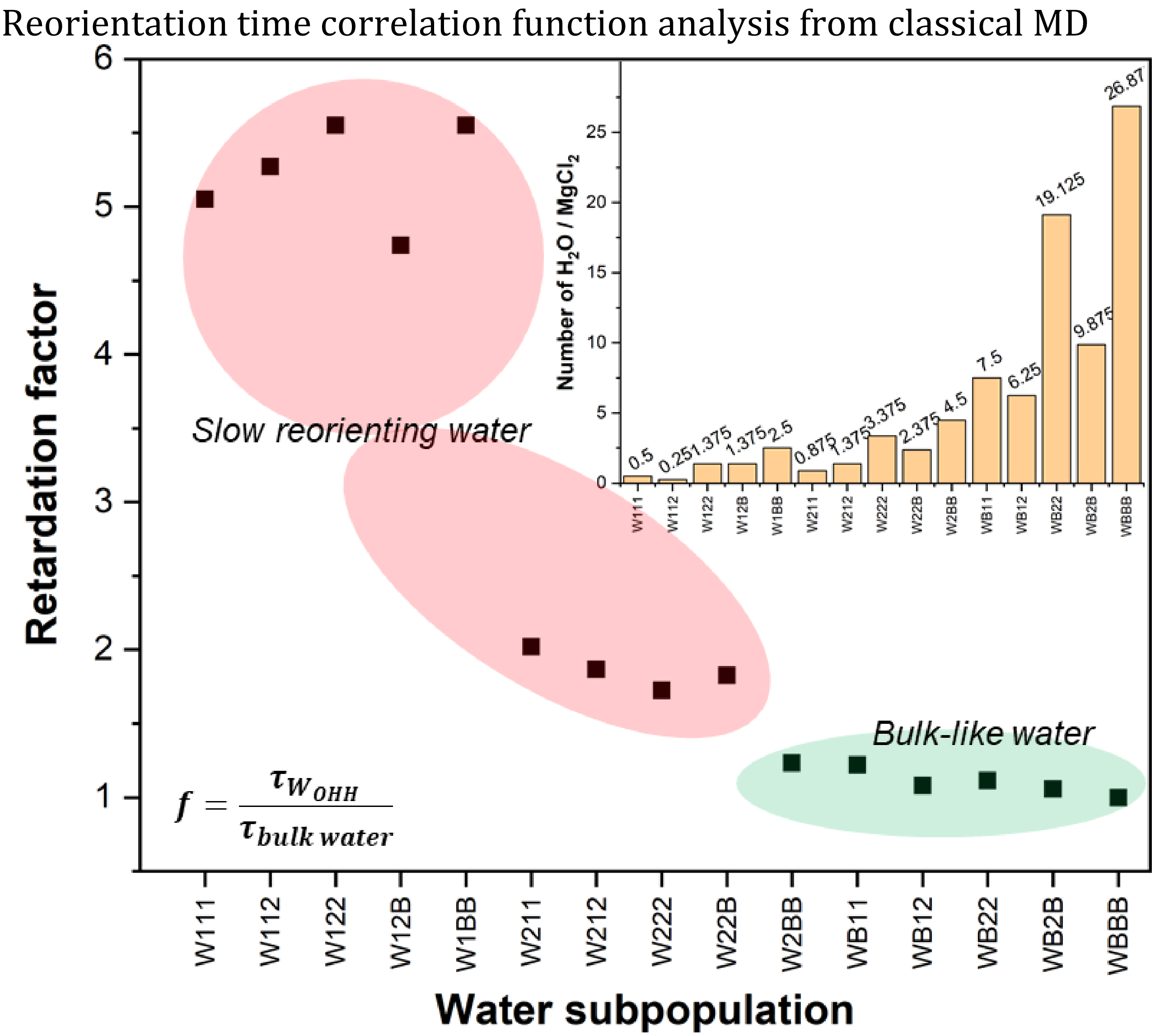

FIGURE S15. Retardation factor for the reorientation relaxation time of the water subpopulations obtained from classical MD simulation of the 0.6 mol.kg^{-1} solution using the Duboue-Dijon forcefield.[15] *Inset*: number of water molecules per $MgCl_2$ units in each subpopulation.

Connection between single-water molecule reorientational dynamics and dielectric relaxation spectroscopy measurements

The frequency-dependent dielectric constant (ε) is obtained from the Fourier-Laplace transform of the time-derivative of dielectric decay function (ϕ):

$$\frac{\varepsilon(\omega)-1}{\varepsilon(0)-1}=\int_0^{\infty}\left(-\frac{d\phi(t)}{dt}\right)e^{-i\omega t}\,dt \tag{1}$$

where ϕ is given by the normalized autocorrelation function of the total dipole moment $\vec{P}$:[16]

$$\phi(t)=\frac{<\vec{P}(t)\cdot\vec{P}(0)>}{<P(0)^2>} \tag{2}$$

For an ensemble of (water) molecules, each characterised by an unit vector $\vec{\mu}$ defining the orientation of the molecular dipole moment, the total dipole moment is given by:

$$\vec{P}=\sum_i \vec{\mu}_i \tag{3}$$

Using this expression for total dipole moment in **Eq. 2**, the dielectric decay function can be written in terms of autocorrelation and cross-correlation of the molecular dipole:

$$\phi(t)=\frac{<\vec{\mu}_i(0)\cdot\vec{\mu}_i(t)>+<\vec{\mu}_i(0)\cdot\sum_{j\neq i}\vec{\mu}_j(t)>}{<\mu_i(0)^2>+<\vec{\mu}_i(0)\cdot\sum_{j\neq i}\vec{\mu}_j(0)>}=\frac{\psi(t)+\gamma(t)-1}{\gamma(0)} \tag{4}$$

where $\psi(t)$ denotes the autocorrelation function of the molecular dipole moments,

$$\psi(t)=\frac{<\vec{\mu}_i(0)\cdot\vec{\mu}_i(t)>}{<\mu_i(0)^2>} \tag{5}$$

and $\gamma(t)$ denotes the multi-molecular dipole cross-correlation function,

$$\gamma(t)-1=<\vec{\mu}_i(0)\cdot\sum_{j\neq i}\vec{\mu}_j(t)>/<\mu_i(0)^2> \tag{6}$$

Let us assume that the auto-correlation function of the molecular dipole $\psi(t)$ and the auto-correlation of the total dipole $\phi(t)$ are governed by one correlation time:

$$\tau_{\phi}=-\phi(t)/\{d\phi(t)/dt\} \tag{7}$$

$$\tau_{\psi}=-\psi(t)/\{d\psi(t)/dt\} \tag{8}$$

where τ_{ϕ} and τ_{ψ} are the *macroscopic* decay function and *molecular* dipole moment decay function, respectively. The expression connecting these two functions has been derived by Kivelson and Madden:[17]

$$\tau_{\phi} = \tau_{\psi}\left[1 + \frac{< \overrightarrow{\mu_{\iota}(0)} \cdot \sum_{i \neq j} \overrightarrow{\mu_1(t)} >}{< \mu_i(0)^2 >} > \right] = \tau_{\psi}\gamma(0) \tag{9}$$

Since the steady function $\gamma(0)$ corresponds to the Kirkwood dipole orientation correlation factor, g_K,[18] we can simply rewrite **Eq. 8** as:

$$\tau_{\psi} = \tau_{\phi}/g_K \tag{10}$$

In our study, we have found that the relaxation time of the molecular dipole correlation function τ_{ψ} of certain water subpopulation are retarded (2 to 6 times slower) compared to bulk-like water molecules. On the other hand, τ_{ϕ} is the same for all water subpopulation because the macroscopic relaxation time is obtained from the total dipole auto-correlation. it follows from **Eq. 9** that water subpopulations with a retarded reorientation dynamics have a lower Kirkwood factor compare to the bulk-like water. Based on the conclusion made by Rinne et al. on single water versus collective water effects on ion-specific reorientation water dynamics,[19] the presence of an ion perturbs the cooperative structure of water in the subpopulations with a retarded reorientation dynamics because ion because of lower Kirkwood dipole orientation correlation factor. The lower dipole correlation factor of the water subpopulation with retarded dipole correlation makes this water subpopulation less contribute to bulk relaxation dynamics. This interpretation is also corroborated by the reduction of multi-molecular dipole cross-correlation contribution in the dielectric loss spectrum calculated, as suggested by Rinne et al.[19] Also, in the dielectric relaxation spectroscopy, the hydration number is calculated from the dielectric loss spectrum of electrolyte solution compare to bulk water. This leads to a decrease in the dielectric loss spectrum (static depolarization) and from static depolarization we can get hydration number.

To summarize, the hydration number computed from the water reorientation dynamic analysis of ab initio and classical MD simulations connects to THz-DR experimental values as follow: the presence of ions in solution can cause the single-molecule reorientation dynamic of some water subpopulations to be slower than bulk water; water subpopulation with retarded reorientational dynamics have lower correlation with other water molecules compared to bulk-like water; in these water subpopulation the multi-molecular dipolar correlation contribution to the dielectric loss spectrum is decreased; as a result, the dielectric loss spectrum, which is measured by DRS is lower than that of bulk water.

References

[1] T. T. Duignan, G. K. Schenter, J. L. Fulton, T. Huthwelker, M. Balasubramanian, M. Galib, M. D. Baer, J. Wilhelm, J. Hutter, M. Del Ben, X. S. Zhao, C. J. Mundy, *Phys. Chem. Chem. Phys.* **2020**, *22*, 10641–10652.

[2] M. Galib, M. D. Baer, L. B. Skinner, C. J. Mundy, T. Huthwelker, G. K. Schenter, C. J. Benmore, N. Govind, J. L. Fulton, *J. Chem. Phys.* **2017**, *146*, 84504.

[3] V. A. Glezakou, Y. Chen, J. L. Fulton, G. K. Schenter, L. X. Dang, *Theor. Chem. Acc.* **2006**, *115*, 86–99.

[4] S. Roy, V. S. Bryantsev, *J. Phys. Chem. B* **2018**, *122*, 12067–12076.

[5] D. Di Tommaso, N. H. de Leeuw, *Cryst. Growth Des.* **2010**, *10*, 4292–4302.

[6] K. M. Callahan, N. N. Casillas-Ituarte, M. Roeselová, H. C. Allen, D. J. Tobias, *J. Phys. Chem. A* **2010**, *114*, 5141–5148.

[7] I. Bakó, J. Hutter, G. Pálinkás, *J. Chem. Phys.* **2002**, *117*, 9838–9843.

[8] J. L. Fulton, S. M. Heald, Y. S. Badyal, J. M. Simonson, *J. Phys. Chem. A* **2003**, *107*, 4688–4696.

[9] A. Chandra, *Phys. Rev. Lett.* **2000**, *85*, 768–771.

[10] J. Åqvist, *J. Phys. Chem.* **1990**, *94*, 8021–8024.

[11] E. Duboué-Dijon, P. E. Mason, H. E. Fischer, P. Jungwirth, *J. Phys. Chem. B* **2018**, *122*, 3296–3306.

[12] S. Pezzotti, D. R. Galimberti, M.-P. Gaigeot, *Phys. Chem. Chem. Phys.* **2019**, *21*, 22188–22202.

[13] S. Pezzotti, D. R. Galimberti, Y. R. Shen, M. P. Gaigeot, *Minerals* **2018**, *8*, 1–16.

[14] R. Khatib, E. H. G. Backus, M. Bonn, M. J. Perez-Haro, M. P. Gaigeot, M. Sulpizi, *Sci. Rep.* **2016**, *6*, 1–10.

[15] E. Duboue-Dijon, P. E. Mason, H. E. Fischer, P. Jungwirth, *J. Phys. Chem. B* **2017**,

122, acs.jpcb.7b09612.

[16] D. van der Spoel, P. J. van Maaren, H. J. C. Berendsen, *J. Chem. Phys.* **1998**, *108*, 10220–10230.

[17] D. Kivelson, P. Madden, *Mol. Phys.* **1975**, *30*, 1749–1780.

[18] U. Kaatze, *J. Solution Chem.* **1997**, DOI 10.1007/BF02768829.

[19] K. F. Rinne, S. Gekle, R. R. Netz, *J. Phys. Chem. A* **2014**, *118*, 11667–11677.
